# DNA barcoding of *Aristolochia* plants and development of species-specific multiplex PCR to aid HPTLC in ascertainment of *Aristolochia* herbal materials

**DOI:** 10.1371/journal.pone.0202625

**Published:** 2018-08-20

**Authors:** Piroonrat Dechbumroong, Surattana Aumnouypol, Jessada Denduangboripant, Suchada Sukrong

**Affiliations:** 1 Research Unit of DNA Barcoding of Thai Medicinal Plants, Department of Pharmacognosy and Pharmaceutical Botany, Chulalongkorn University Drug and Health Products Innovation Promotion Center, Faculty of Pharmaceutical Sciences, Chulalongkorn University, Bangkok, Thailand; 2 Department of Biology, Faculty of Sciences, Chulalongkorn University, Bangkok, Thailand; Chinese Academy of Medical Sciences and Peking Union Medical College, CHINA

## Abstract

The anecdotal evidence is outstanding on the uses of *Aristolochia* plants as traditional medicines and dietary supplements in many regions of the world. However, herbal materials derived from *Aristolochia* species have been identified as potent human carcinogens since the first case of severe renal disease after ingesting these herbal preparations. Any products containing *Aristolochia* species have thus been banned on many continents, including Europe, America and Asia. Therefore, the development of a method to identify these herbs is critically needed for customer safety. The present study evaluated DNA barcoding of the *rbc*L, *mat*K, ITS2 and *trn*H-*psb*A regions among eleven *Aristolochia* species collected in Thailand. Polymorphic sites were observed in all four DNA loci. Among those eleven *Aristolochia* species, three species (*A*. *pierrei*, *A*. *tagala* and *A*. *pothieri*) are used as herbal materials in Thai folk medicine, namely, in Thai “Krai-Krue”. “Krai-Krue” herbs are interchangeably used as an admixture in Thai traditional remedies without specific knowledge of their identities. A species-specific multiplex PCR based on nucleotide polymorphisms in the ITS2 region was developed as an identification tool to differentiate these three *Aristolochia* species and to supplement the HPTLC pattern in clarifying the origins of herbal materials. The combination of multiplex PCR and HPTLC profiling achieves accurate herbal identification with the goal of protecting consumers from the health risks associated with product substitution and contamination.

## Introduction

The genus *Aristolochia*, a member of the family Aristolochiaceae, consists of approximately 500 species and is widely distributed in tropical and subtropical areas including Asia, Africa, Europe and the Americas [1, 2]. Several *Aristolochia* species have been medicinally used in many traditional drug formulas and dietary supplements in many parts of the world. In China, *A*. *fangchi* has been used as an antirheumatic and a diuretic [3], while *A*. *manshuriensis* has been used for treating problems relating to the urine and bladder problems [3]. *A*. *gigantea* and *A*. *anguicida* have been used as abortifacients and to counteract snake venoms in Brazil [4], respectively. *A*. *grandiflora* was used in Panama to treat snakebites, gastrointestinal disorders and fever, similar to the uses of *A*. *ringens* in South America [3]. In addition, *A*. *tagala* and *A*. *indica* were used as emmenagogues, antirheumatics and snakebite remedies in India [5]. *Aristolochia pothieri* has been used for longevity in Thailand [3]. Various bioactivities of *Aristolochia* plants have been continuously reported, such as bactericidal activity by *A*. *mollissima* [6], cyclin-dependent kinase 2 (CDK2) enzyme inhibition by *A*. *manshuriensis* [7] and strong inhibition of L-amino acid oxidase by *A*. *indica* [8].

Plants in the genus Aristolochia produce many types of aristolochic acids (AAs), for example, AA I, II, III, IV and IV, at various concentrations. Two major chemical constituents are aristolochic acid I (AAI) and aristolochic acid II (AAII). AAI is commonly found at higher concentration than AAII [9], making this compound suitable for chemical analysis. Two major chemical constituents are aristolochic acid I (AAI) and aristolochic acid II (AAII). AAI is commonly found at higher concentration than AAII [9]. AAI and AAII have been classified as kidney-targeting carcinogenic substances since the first case of severe renal disease was identified in a Belgian women who took slimming pills containing *Aristolochia fangchi* instead of *Stephania tetrandra* in the 1990s [10]. The misidentification of herbal materials as a cause of toxicity to consumers has been reported as a global concern [11]. Digestion of herbal remedies containing *Aristolochia* plants clearly causes severe toxic effects including renal fibrosis, irreversible nephropathy, renal failure and kidney cancer [12] through a DNA adduct mechanism, due to AAs and their metabolites [13–14]. Since 2007, *Aristolochia-*containing products have been banned in the United States of America, Australia, Europe and Asia [9]. In 2013, the National Drug Committee of Thailand also legally issued an order to manufacturers to remove *Aristolochia* herbal materials from all formulas [15–16]. Despite being banned in many countries, a number of AA-containing products still appear to be offered for sale through internet and local dispensaries.

To assure the correct authentication of natural materials, many identification tools have been developed [17]. The determination of the presence of AAI by chemical profiling, such as high-performance thin-layer chromatography (HPTLC) [18–21], liquid chromatography with ultraviolet detection (LC/UV) [18, 20, 22], and liquid chromatography coupled with mass spectrometry (LC/MS) [18, 23], is among the useful procedures for assessing suspicious products. Following the European Pharmacopoeia and British Pharmacopoeia, an HPTLC pattern using AAI as the standard marker has been used as a reference for the screening of suspected herbal products since 2012 [18, 24]. However, chemical patterns have some limitations in authenticating plant materials; for example, chemical constituents can be altered due to agricultural method, harvest time and weather [25]. In addition, microscopic and macroscopic approaches, which are primary plant authentication methods, require expert practitioners, and sometimes, the botanically unique characters of the plants are not intact in the crude drugs [26]. Recently, many molecular biological technologies have been developed as useful tools for DNA analysis of medicinal plants [27–28]. DNA barcoding is the one of the latest tools and represents a gold-standard molecular identification technique. This method can provide accurate identification of plant samples that are not distinguishable by morphology or by name, and it is included in the latest edition of the Pharmacopoeia of the People’s Republic of China and its online supplementary note [29].

DNA barcodes based on five candidate regions (*rbc*L, *mat*K, ITS, *trn*H-*psb*A and *trn*L-*trn*F) have already been established for the identification of the *Aristolochia* medicinal plants used in China, including *A*. *fangchi*, *A*. *manshuriensis*, *A*. *contorta* and *A*. *debilis* [30]; *A*. *mollissima* [20]; and *A*. *californica*, *A*. *championii*, *A*. *contorta*, *A*. *debilis*, *A*. *heterophylla* and *A*. *kaempferi* [31]. However, the limited number of *Aristolochia* plants in specific regions has restricted the development of rapid molecular identification techniques for these plants.

Among the numerous *Aristolochia* species found in Thailand, three *Aristolochia* species, *A*. *pothieri* [32], *A*. *pierrei* [33–34] and *A*. *tagala* [34] have been medicinally used in Thai folk medicine under the common name “Krai-Krue”. Beside these three *Aristolochia* species, *Raphistemma pulchellum* [35], *Gymnopetalum integrifolium* [36], and *Jasminum* spp. [37] has been reported as substitutes for “Krai-Krue”. In their crude drug form, the dried roots of “Krai-Krue” are not identifiable by morphological characters; organoleptic tests and chemical patterns result in the interchangeable use of multiple *Aristolochia* species or other plant species. Thus, we aimed to establish core DNA barcodes (*rbc*L, *mat*K, ITS2 and *trn*H-*psb*A) for eleven *Aristolochia* species found in Thailand and contribute this information to the plant DNA barcode reference library. DNA barcoding of ITS2 was used to develop a species-specific molecular marker for identification of the botanical origin of “Krai-Krue” herbs. HPTLC profiling was combined with this species-specific molecular marker to investigate the identities of crude drug samples from various local dispensaries. Phylogenetic relationships based on the nucleotide sequence data sets of *rbc*L, *mat*K and ITS2 were estimated and discussed.

## Materials and methods

### Plant materials

Thirty-eight specimens of eleven *Aristolochia* taxa were collected from various regions of Thailand. Five specimens of different species considered as sources of “Krai-Krue” herb (*Raphistemma pulchellum*, *Gymnopetalum integrifolium*, *Jasminum sambac*, *Jasminum adenophyllum* and *Jasminum sp*.) were included in the study (Table 1). All specimens were identified by Associate Professor Thatree Phadungcharoen at the Department of Pharmacognosy and Pharmaceutical Botany, Faculty of Pharmaceutical Sciences, Chulalongkorn University, Thailand. Herbarium specimens were prepared and kept at the Museum of Natural Medicines, Faculty of Pharmaceutical Science, Chulalongkorn University. Seven commercial crude drug samples (C1-C7) claiming to be “Krai-Krue” were purchased from local dispensaries (Table 1).

**Table 1:** Plant materials and crude drugs used in this study.

Sample	Place of collection (Thailand, Province)	Voucher number	GenBank accession number			
			*rbc*L	*mat*K	*trn*H-*psb*A	ITS2
***Aristolochia* species**						
***A*. *pierrei* Lecomte**	Sakon Nakhon	MUS-5407	-	-	-	-
	Sakon Nakhon	MUS-5408	-	-	-	-
	Sakon Nakhon	MUS-5409	KP998768	KP998782	KP998810	KP998796
	Sakon Nakhon	MUS-5410	-	-	-	-
	Sakon Nakhon	MUS-5411	-	-	-	-
***A*. *pothieri* Pierre ex Lecomte**	Bangkok	MUS-5374	KP998769	KP998783	KP998811	KP998797*
	Bangkok	MUS-5381	-	-	-	-
	Bangkok	MUS-5382	-	-	-	-
	Bangkok	MUS-5402	-	-	-	-
	Bangkok	MUS-5403	-	-	-	-
***A*. *tagala* Cham.**	Chiang Mai	MUS-5400	KP998772	KP998786	KP998814	KP998800
	Bangkok	MUS-5450	-	-	-	-
	Bangkok	MUS-5451	-	-	-	-
***A*. *anguicida* Jacq.**	Chiang Mai	MUS-5405	KP903720	KP998777	KP998805	KP998791
	Chiang Mai	MUS-5406	-	-	-	-
***A*. *gigantea* Mart. et Zucc.**	Bangkok	MUS-5393	KP998764	KP998778	KP998806	KP998792
	Nakhon Pathom	MUS-5376	-	-	-	-
	Bangkok	MUS-5377	-	-	-	-
	Chiang Mai	MUS-5396	-	-	-	-
	Bangkok	MUS-5394	-	-	-	-
	Bangkok	MUS-5395	-	-	-	-
	Chiang Mai	MUS-5397	-	-	-	-
***A*. *grandiflora* Sw.**	Lampang	MUS-5391	KP998765	KP998779	KP998807	KP998793*
	Phitsanulok	MUS-5379	-	-	-	-
	Petchabun	MUS-5390	-	-	-	-
	Lampang	MUS-5392	-	-	-	-
	Phitsnulok	MUS-5380	-	-	-	-
***A*. *kerrii* Craib**	Chiang Mai	MUS-5413	KP998766	KP998780	KP998808	KP998794
	Chiang Mai	MUS-5415	-	-	-	-
***A*. *littoralis* D. Parodi**	Bangkok	MUS-5404	KP998767	KP998781	KP998809	KP998795
	Bangkok	MUS-5452	-	-	-	-
	Phetchaburi	MUS-5453	-	-	-	-
	Phetchaburi	MUS-5454	-	-	-	-
***A*. *ringens* Vahl**	Bangkok	MUS-5375	KP998770	KP998784	KP998812	KP998798
	Bangkok	MUS-5383	-	-	-	-
	Nakhon Pathom	MUS-5384	-	-	-	-
	Nakhon Pathom	MUS-5385	-	-	-	-
	Chiang Mai	MUS-5387	-	-	-	-
	Chiang Mai	MUS-5388	-	-	-	-
	Bangkok	MUS-5389	-	-	-	-
	Bangkok	MUS-5412	-	-	-	-
***A*. *tentaculata* Schmidt in Fedde**	Bangkok	MUS-5398	KP998773	KP998787	KP998815	KP998801
	Bangkok	MUS-5455	-	-	-	-
	Phetchaburi	MUS-5456	-	-	-	-
***Aristolochia* sp.**	Bangkok	MUS-5399	KP998771	KP998785	KP998813	KP998799
**Other sources of “Krai-Krue”**						
***Raphistemma pulchellum* Wall**	Bangkok	MUS-5414	-	-	-	MG870094
***Gymnopetalum integrifolium* Kurz.**	Sakaeo	MUS-5415	-	-	-	MG870090
	Phetchaburi	MUS-5416	-	-	-	-
***Jasminum sambac* (L.) Aiton**	Bangkok	MUS-5417	-	-	-	MG870093
***Jasminum adenophyllum* Wall. ex C.B.Clarke**	Bangkok	MUS-5418	-	-	-	MG870091
***Jasminum* sp.**	Bangkok	MUS-5419	-	-	-	MG870092
**Herbs**						
**Krai-Krue 1**	Bangkok	C1	-	-	-	-
**Krai-Krue 2**	Bangkok	C2	-	-	-	-
**Krai-Krue 3**	Nakhon Si Thammarat	C3	-	-	-	-
**Krai-Krue 4**	Phetchaburi	C4	-	-	-	-
**Krai-Krue 5**	Ayutthaya	C5	-	-	-	-
**Krai-Krue 6**	Bangkok	C6	-	-	-	-
**Krai-Krue 7**	Bangkok	C7	-	-	-	-

*Sequences consist of ITS1 and ITS2 regions.

### HPTLC screening test for AAI

Screening for AAs in herbs and herbal products by HPTLC pattern has been recommended by the European Pharmacopoeia and British Pharmacopoeia since 2012 [18, 24]. The test solution was prepared by extracting 2 g of the powdered herbal drug with 10 mL of anhydrous formic acid (Merck, Germany)-water (Merck, Germany)-methanol (Merck, Germany) (1:9:40, V/V/V), and then sonicating the mixture at room temperature for 10 min and centrifuging at 14,000 rpm for 5 min. The clear solution was used as the test solution for 1 μL (for C1-C5) and 20 μL (for C6-C7 and all remedies) as bands of 8 mm. An upper layer of a mixture of anhydrous formic acid (Merck, Germany)-water (Merck, Germany)-ethyl acetate (Merck, Germany)-toluene (Merck, Germany) (1:1:10:20, V/V/V/V) was used as the mobile phase. The plate was sprayed with a 100 g/L solution of stannous chloride (Merck, Germany) in dilute hydrochloric acid (Merck, Germany) until slightly wet and then heated at 100°C for 1 min. AAI (Sigma-Aldrich, USA) was used as a standard at concentrations of 2 and 5 ppm at 20 μL. All materials and reagents were of analytical grade. TLC was performed using an HPTLC silica gel 60 F_254_ glass plate 20x10 cm. The solvent fronts of the mobile phases were allowed to ascend 8 cm above the line of sample application. The chromatograms were observed under long (365 nm) ultraviolet wavelengths.

### Genomic DNA extraction

Total genomic DNA was extracted from 80 to 100 mg of fresh leaves of the plants or from 15 to 25 mg of dried commercially available crude drug samples. DNA was extracted by a DNeasy Plant Mini Kit (Qiagen, Germany) according to the manufacturer's protocol. DNA quality and quantity were determined by agarose gel electrophoresis; the gels were stained with ethidium bromide and visualized under UV light.

### PCR amplification and electrophoresis

The *rbc*L, ITS2, and *trn*H-*psb*A of *Aristolochia* were amplified by amplification primers (Table 2). Nucleotide sequences of the *trn*K-*mat*K regions of *A*. *pierrei* (accession number DQ296649), *A*. *grandiflora* (accession number DQ532052), and *A*. *gigantea* (accession numbers JX485569 and DQ882187) were retrieved from GenBank for primer design. New primers, matK-Aris-F1, matK-Aris-F2 and matK-Aris-R1 (Table 2), were used to amplify the complete *mat*K sequence of the genus *Aristolochia*.

**Table 2:** Primers used for DNA barcode generation.

DNA locus	Sequencing primer	Sequence (5'→3')	References
***rbc*L**	rbcL_aF*	ATG TCA CCA CAA ACA GAG ACT AAA GC	[38]
	rbcL-Aris-327R	TTC AAA AAG GTC TAA AGG GTA AGC	In this study.
	rbcL_636F	GCG TTG GAG AGA TCG TTT CT	[39]
	rbcL_R23*	TTT TAG TAA AAG ATT GGG CCG	[39]
***mat*K**	matK-Aris-F1*	ATC CCC TAT TCC TTC AGT TCA A	In this study.
	matK-Aris-F2*	CCT TGT TTT GAC TGT ATC GCA C	In this study.
	matK-Aris-F458	ATA CCC CAC CCC ATC CAT CTG	In this study.
	matK-Aris-F967	CAC TTG TGG TCT CAA CCG GG	In this study.
	matK-Aris-R1*	GCA CAC GGC TTT CCC TAT G	In this study.
***trn*H-*psb*A**	psbAF*	GTT ATG CAT GAA CGT AAT GCT C	[31]
	trnHR*	CGC GCA TGG TGG ATT CAC AAA TC	[31]
**ITS2**	ITS1*	TCC GTA GGT GAA CCT GCG G	[40]
	ITS3*	GCA TCG ATG AAG AAC GCA GC	[40]
	ITS4*	TCC TCC GCT TAT TGA TAT GC	[40]
	ITS-Aris-390F	AAT TGC AGA ATC CCG CGA AC	In this study.

* Primers used as sequencing primer and amplification primer.

The PCR amplification was performed in 50 μL of reaction mixture consisting of 5X PCR buffer, 25 mM MgCl_2,_ 2.5 mM each dNTP, 10 mM each primer, 5U GoTaq® Flexi DNA polymerase (Promega, USA), and 10–100 ng of total DNA as a template. PCR amplifications were carried out in a C1000™ Thermal Cycler (Bio-Rad, USA) using cycling conditions of 96°C for 3 min; followed by 30 cycles of 96°C for 1 min, 55°C for 1 min and 72°C for 2 min (for *rbc*L and *mat*K) and 45 sec (for ITS2 and *trn*H-*psb*A); and a final extension at 72°C for 10 min. The amplified products were detected by 1.2% agarose gel electrophoresis in 1X TAE buffer and were visualized by ethidium bromide staining.

### Sequence analysis

The sequencing process was performed by capillary sequencing (AIT Biotech, Singapore) with sequencing primers for each region (Table 2). The sequences were aligned, edited and analysed using BioEdit Sequence Alignment Editor Version 7.2.5 [41]. The obtained sequences were assembled to create consensus sequences using the DNASTAR^®^ (Version 8.0.2) program (USA). The sequences were then submitted to the DDBJ/EMBL/GenBank nucleotide sequence databases, and their accession numbers (Table 1).

### Multiplex PCR technique for discrimination of the three medicinal *Aristolochia* species named “Krai-Krue”

**Fig 1:**
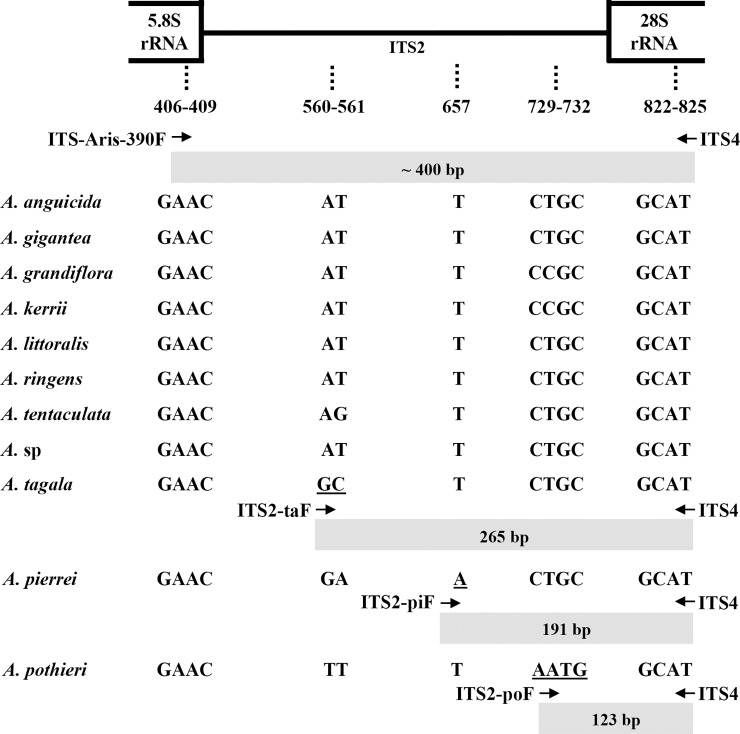
A schematic diagram of the ITS2 region in eleven *Aristolochia* species and related species. Dotted lines represent the positions of diagnostic nucleotides. Underlined nucleotides represent the species-specific 3' ends of the primers. Arrows indicate the orientations and approximate positions of the species-specific primers. The grey boxes represent the PCR products from multiplex PCR.

The ITS2 nucleotide sequences of the three medicinal “Krai-Krue” species were aligned to identify their unique nucleotide polymorphisms. Based on those sites, three species-specific primers (Table 3) were designed. A primer pair, ITS-Aris-390F and ITS-4, was used as a positive control specific to all species (Fig 1). Pentaplex PCR amplification was performed in 50 μL of reaction mixture consisting of 5X PCR buffer, 25 mM MgCl_2,_ 2.5 mM each dNTP, 10 mM each primer, 5U GoTaq® Flexi DNA polymerase (Promega, USA), and 10–100 ng of total DNA as a template. PCR amplifications were carried out in a C1000™ Thermal Cycler (Bio-Rad, USA) using cycling conditions of 96°C for 3 min; followed by 30 cycles of 96°C for 1 min, 52°C for 1 min and 72°C for 30 sec; and a final extension at 72°C for 10 min. The amplified products were detected by 1.7% agarose gel electrophoresis in 1X TAE buffer and were visualized by ethidium bromide staining.

**Table 3:** Species-specific primers used in multiplex PCR. Underlined and italicised nucleotides represent the species-specific 3' ends of the primers.

Primer	Species specificity	Sequence (5'→3')
ITS2-poF	*A*. *pothieri*	GCC GCG AGG ACC C*AA TG*
ITS2-piF	*A*. *pierrei*	GAC TAC TGG TGG CTC CAC GC*A** *
ITS2-taF	*A*. *tagala*	GGC GGG GGC GAG CAG *GC*
ITS-Aris-390F	Internal amplification control	AAT TGC AGA ATC CCG CGA AC
ITS4	Internal amplification control	TCC TCC GCT TAT TGA TAT GC

### Phylogenetic analysis

A combined data matrix of the *rbc*L and *mat*K chloroplast DNA sequences was aligned using the CLUSTAL X 2.1 multiple alignment program [42]. A phylogenetic tree using the maximum likelihood (ML) method was constructed with the MEGA version 6 program [43]. The ML analysis was performed using the Kimura-2-parameter nucleotide substitution model with the nearest neighbour interchange searching strategy. In addition to the sequences of eleven *Aristolochia* taxa, those of *Thottea borneensis* (GenBank accession numbers *rbc*L: AB205604 and *mat*K: JN415668) and *Thottea tomentosa* (*rbc*L: AB205606 and *mat*K: JN415674), which are also members of the family Aristolochiaceae, were retrieved from the GenBank nucleotide database and used as outgroups. A bootstrapping statistical analysis was performed (with 1,000 replicates) to estimate the accuracy of the topology of the ML tree found. Moreover, an alignment of the ITS2 sequences of the nuclear genomes of *Aristolochia* were also prepared and analysed phylogenetically with the same Kimura-2 model and ML method. The ITS2 sequences of *Asarum caudatum* (GenBank accession number KJ888492.1) and *Asarum yakusimense* (AB699853.1), additional plants in the Aristolochiaceae, were used as outgroups.

## Results and discussion

### DNA barcodes of the eleven *Aristolochia* species

DNA barcoding of *Aristolochia* plants has been investigated and performed as an effective identification tool for customer safety [20, 23, 30, 31]. However, the limited number of DNA sequences from these plants in any specific region has restricted the development of rapid DNA barcoding identification for *Aristolochia* plants. In this study, all four candidate DNA regions including *rbc*L, *mat*K, ITS2 and *trn*H-*psb*A performed very well in eleven *Aristolochia* plant samples found in Thailand. The nucleotide sequences of full-length *rbc*L, full-length *mat*K and ITS sequences and partial *trn*H-*psb*A sequences of *Aristolochia* plants were established (Table 1). The degree of sequence variation among the *Aristolochia* samples was ranked as follows: ITS2 > *trn*H-*psb*A > *mat*K > *rbc*L. The large insertions/deletions in ITS2 caused high variation (28.98%) among the eleven *Aristolochia* species, much higher than those of *trn*H-*psb*A (11.56%), *mat*K (11.15%) and *rbc*L (3.29%) (Table 4).

**Table 4:** Properties of the four DNA barcoding regions of *Aristolochia* plants.

Species		*rbc*L			*mat*K			*trn*H-*psb*A			ITS2	
	Length (bp)	GC content (%)	Variation (%)	Length (bp)	GC content (%)	Variation (%)	Length (bp)	GC content (%)	Variation (%)	Length (bp)	GC content (%)	Variation (%)
*A*. *anguicida*	1428	44.96		1539	34.50		300	40.33		399	69.42	
*A*. *gigantea*	1428	45.03		1554	34.23		305	40.00		461	65.73	
*A*. *grandiflora*	1428	45.03		1524	33.92		308	38.96		751*	64.98*	
*A*. *kerrii*	1428	44.40		1527	34.51		319	39.81		360	76.39	
*A*. *littoralis*	1428	45.03		1539	34.18		305	39.67		437	67.73	
*A*. *pierrei*	1428	45.10	3.29	1518	34.58	11.15	315	40.32	11.56	379	70.71	28.98
*A*. *pothieri*	1428	44.82		1524	34.19		369	34.96		696*	70.69*	
*A*. *ringens*	1428	44.89		1548	34.75		305	40.00		432	68.06	
*A*. *tagala*	1428	44.96		1518	34.52		318	39.94		378	71.69	
*A*. *tentaculata*	1428	45.03		1524	34.84		310	40.00		436	66.97	
*A*. sp.	1428	44.96		1539	34.44		283	40.28		431	67.95	

*Sequences consist of ITS1 and ITS2 regions.

DNA barcodes with diagnostic polymorphic sites in *Aristolochia* plants can serve as a suitable biomolecular tool for authentication. This method provides complementary information to the results of previous studies, which were based on the plants used in Chinese traditional medicine, for example, *A*. *fangchi*, *A*. *manshuriensis*, *A*. *contorta* and *A*. *debilis* [30]; *A*. *mollissima* [20]; and *A*. *californica*, *A*. *championii*, *A*. *contorta*, *A*. *debilis*, *A*. *heterophylla* and *A*. *kaempferi* [31]. The expansion of the DNA database of these plants will help to alleviate the restrictions of the DNA barcoding method in monitoring crude drugs in the global market. Furthermore, it will be useful for safety control by the herbal industries and regulatory authorities.

The obtained sequences from this study were searched against the published DNA databank. The BLAST outputs suggested that our DNA sequences in *rbc*L and *mat*K coding regions of plant samples were matched to the published DNA sequences. However, a few variations in *trn*H-*psb*A and ITS2 regions between sample sequences and sequences in DNA database have been found.

### Development of species-specific multiplex PCR based on the ITS2 region to aid HPTLC profiling in identification of “Krai-Krue” herbs

“Krai-Krue” is a crude drug used as an ingredient in Thai folk medicinal remedies in small amounts based on the inexplicable Thai traditional medicine belief that a small amount of toxic substances can be neutralized by the other compounds in the remedy [35]. “Krai-Krue” (Table 1) is derived from the dried roots of three *Aristolochia* species, including *A*. *pothieri* [32], *A*. *pierrei* [33–34], and *A*. *tagala* [34], and substitute species, including *Raphistemma pulchellum* [35], *Jasminum sambac*, *Jasminum adenophyllum*, *Jasminum* sp. [37], and *Gymnopetalum integrifolium* [36]. A previous report has stated that the various botanical sources of “Krai-Krue” can lead to confusing usage of crude drugs and may cause nephrotoxicity due to the ingestion of raw *Aristolochia* material [12]. For the safety of consumers, the National Drug Committee of Thailand legally issued an order to manufacturers to remove “Krai-Krue” from all formulas within one year after April 19th, 2013 [15–16]. However, *Aristolochia*-containing products are still present in local herbal markets. Thus, a fast and accurate tool to identify herbal materials is needed.

Based on its discriminatory power at the species level, the ITS2 region is recommended as an additional barcode for plant identification with modest universality and sequence quality compared to those of the combined *rbc*L and *mat*K sequence and *trn*H-*psb*A sequence [44]. The ITS2 region is the most frequently used for identification of herbal materials [23, 27, 45]; the high degree of sequence variation and short amplicons of ITS2 in eleven *Aristolochia* species were beneficial to the success of PCR amplification, especially in commodity samples with poor DNA integrity because of the post-harvest processing methods and manufacturing protocols for these herbs.

An ITS2 multiple sequence alignment of these eleven *Aristolochia* species and substitute species was performed to identify polymorphic sites in the three *Aristolochia* species used as “Krai-Krue”. Other species used as sources of “Krai-Krue” (Table 1) were included to verify that the PCR amplicon pattern could discriminate the suspected species correctly. From the present GenBank database, only two ITS2 sequences of *A*. *tagala* (KP763869.1 and KP763864.1) were retrieved as extensions for species identification; no information was available regarding the ITS2 sequences of *A*. *pierrei* and *A*. *pothieri*. Although within-species heterogeneity (two bases’ difference) was found in *A*. *tagala*, a molecular marker was developed from the consensus sequence for wide application. Nucleotide polymorphisms in the three *Aristolochia* species were diagnosed at alignment positions 560–561, 657 and 729–732 (Fig 1).

**Fig 2:**
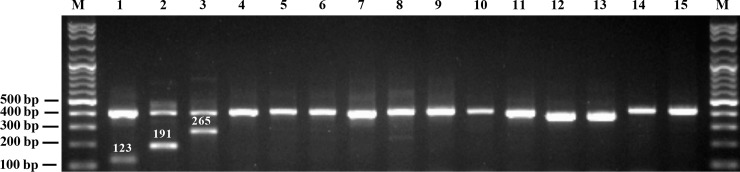
Image of PCR products generated with a set of species-specific PCR primers in the ITS2 region of “Krai-Krue” herbs as detected by 1.7% agarose gel electrophoresis. Lane 1: *Aristolochia pothieri*; lane 2: *A*. *pierrei*; lane 3: *A*. *tagala*; lane 4: *A*. *ringens*; lane 5: *A*. *kerrii*; lane 6: *A*. *littoralis*; lane 7: *A*. *grandiflora*; lane 8: *A*. *gigantea*; lane 9: *A*. *tentaculata*; lane 10: *A*. *anguicida*; lane 11: *Gymnopetalum integrifolium*; lane 12: *Raphistemma pulchellum*; lane 13: *Jasminum sp*.; lane 14: *J*. *sambac*; lane 15: *J*. *adenophyllum* and M: VC 100-bp plus DNA ladder.

Nucleotide polymorphisms in the ITS2 region were used to design species-specific 3' primer ends. The species-specific primer set, ITS-Aris-390F, ITS2-poF, ITS2-piF, ITS2-taF and ITS4 (Table 3), was used to amplify the ITS2 region to ensure that these primers perfectly matched only the sequences corresponding to the target genes and then confirmed by conventional PCR and agarose gel electrophoresis analysis using genomic DNA from the three *Aristolochia* species and other related species as templates. The amplified products successfully distinguished the three species of “Krai-Krue” herbs from the other botanical samples by the different sizes of the PCR products (Fig 2). *Aristolochia pothieri*, *A*. *pierrei* and *A*. *tagala* had product sizes of 123 bp, 191 bp, and 265 bp, respectively, with a single internal control band (400 bp). Sequence search of the PCR products of the three *Aristolochia* species indicated that they are identical to the sequences of ITS2 of *A*. *pothieri*, *A*. *pierrei* and *A*. *tagala* with accession numbers KP998797, KP998796 and KP998800, respectively.

**Fig 3:**
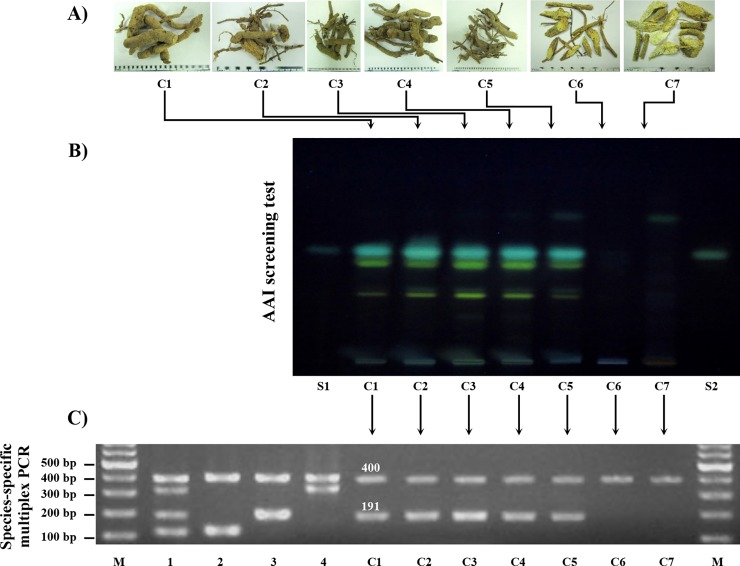
Authentication of seven commercial crude drugs (C1-C7) claimed to be “Krai-Krue” using a chemical screening test and species-specific PCR. (A) HPTLC analysis of herbal samples observed under UV at 365 nm. Lane S1: AAI standard, 2 ppm; lane C1-C7: commercial “Krai-Krue” products (C1-C7); and lane S2: AAI standard, 5 ppm. B) Image of 1.7% agarose gel electrophoresis of species-specific PCR primers amplifying the ITS2 region of *Aristolochia* species used as “Krai-Krue” and seven commercial “Krai-Krue” herbs (C1-C7). (C) DNA marker sizes (M) in bp are indicated. Lane 1: mixed genomic DNA of *Aristolochia* species used as “Krai-Krue”; lane 2: *A*. *pothieri*; lane 3: *A*. *pierrei*; lane 4: *A*. *tagala*; and lanes C1-C7: C1-C7, respectively.

To assay the chemical substances present, HPTLC pattern comparison using AAI as a chemical marker was conducted, as this method has been recommended by the European Pharmacopoeia and British Pharmacopoeia as a screening test to exclude herbal products with AAI present at levels greater than or equal to 2 ppm since 2012 [18]. In this study, *Aristolochia* species were ascertained by a combination of DNA barcoding and HPTLC techniques (Fig 3). Seven commercial crude drugs represented as C1-C7 (Fig 3A) were claimed to be “Krai-Krue” when purchased from local dispensaries. HPTLC profiles were used as a screening test for AAI in the tested herbal materials. AAI at concentrations of 2 and 5 ppm was used as a standard marker. The chromatogram showed the Rf value of the AAI standard at 0.46 with a greenish-blue zone (Fig 3B). The HPTLC profiles of the crude drug extracts were examined. The chromatograms produced by using 1 μL of the C1-C5 extracts showed chromatographic bands at the same Rf value as that of the AAI standard. In contrast, no AAI band was observed in the C6 and C7 extracts (Fig 3B). These results suggested that C1-C5 were AAI-containing products, while C6-C7 were not. Although these chemical fingerprints could identify samples by their major chemical constituents, the botanical sources of these purported “Krai-Krue” samples remained unknown. Many types of DNA-based molecular techniques have been continuously developed to complement chemical profiling as a more accurate and reliable identification tool for herbal materials with minimal cost and time.

Multiplex PCR was applied to test the seven crude drug samples traded as “Krai-Krue” (Fig 3C). The amplification profiles displayed two bands of 191 and 400 bp in C1-C5, which is a pattern characteristic of *A*. *pierrei*, and a single band of 400 bp in C6-C7 (Fig 3C). As the amplification profiles (Fig 3C) and HPTLC patterns (Fig 3B) were consistent, five of these crude drugs (C1-C5) were recognized as *A*. *pierrei*, while two samples (C6-C7) were not *Aristolochia*.

As described above, multiplex PCR based on this DNA barcoding data set can complement HPTLC profiling to achieve reasonable and convenient identification results at low cost and in a short time, with the same discriminatory power at the species level as analytical methods based on sequencing. Future work should focus on the optimization of both PCR conditions and HPTLC systems to enhance sensitivity and specificity of the techniques. A combination of qualitative and quantitative data within a single investigation will be a surrogate analytical method for quality control of *Aristolochia* herbal materials.

### Phylogenetic analysis

**Fig 4:**
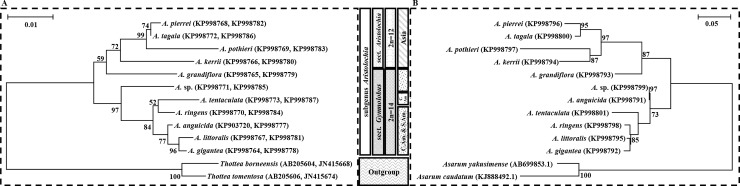
Comparison between the maximum likelihood phylogenetic trees of eleven *Aristolochia* taxa. (A) the combined data set of the *rbc*L and *mat*K genes and (B) the ITS2 nucleotide sequences. Numbers along branches indicate bootstrap percentages (higher than 50%) from the branch-supporting statistical analysis.

The combined dataset of nuclear ITS2 sequences and complete chloroplast *rbc*L and *mat*K sequences of all *Aristolochia* and outgroup samples was analysed. Both ML trees (Fig 4) gave similar topologies and revealed that all *Aristolochia* taxa were monophyletically grouped together with a 100% bootstrap value. The morphological characteristics confirmed that all plant specimens belonged to the subgenus *Aristolochia*. Both molecular phylogenies indicated a division between two major clades, potentially following the previous taxonomic suggestion of González [46], i.e., the Old-World clade, consisting of the section *Aristolochia* Asian species such as *A*. *tagala*, *A*. *pierrei*, *A*. *pothieri* and *A*. *kerrii* (with 72% and 97% bootstrap values in Fig 4A and Fig 4B, respectively), and the New-World clade, consisting of the section *Gymnolobus* from Central and South America (97% and 73% bootstrap values, in Fig 4A and Fig 4B, respectively), with *A*. *ringens*, *A*. *tentaculata*, *A*. *anguicida*, *A*. *littoralis* and *A*. *gigantea* as its members. However, *A*. *grandiflora*, which has been suggested morphologically as another member of the section *Gymnolobus* due to its thyrsoid branching in the inflorescence, was not grouped with other *Gymnolobus* taxa but placed basal to the Old-World clade of section *Aristolochia* in both ML trees.

The phylogenetic analysis results from both the *Aristolochia* chloroplast and nuclear DNA genomes agreed well with the previously published phylogenies of *mat*K [39, 47] and *trn*L-*trn*F [48]. Our phylogenetic results also correlated very well with the morphological characteristics, habitats and chromosome numbers of the plants. Within the section *Aristolochia* Old-World clade, *A*. *pothieri*, *A*. *pierrei* and *A*. *tagala* were strongly grouped together with high bootstrap support values. All these species were previously classified based on their morphology as subsection *Podanthemum* [46]. This subsection has a unique stipitate utricle of the perianth tube and a chromosome number of 2n = 12. *Aristolochia pierrei* and *A*. *tagala* also shared very similar external characteristics. Likewise, these two species later showed similar results from the multiplex PCR analysis, since their amplified nuclear DNA sequence products were of the same size. Nevertheless, *A*. *pothieri* produced a smaller-sized PCR product due to many nucleotide deletions when analysed for discrimination of “Krai-Krue” botanical sources. On the other hand, most of the members of the New-World clade of section *Gymnolobus*, *A*. *tentaculata*, *A*. *anguicida*, *A*. *ringens*, *A*. *littoralis* and *A*. *gigantea*, were from the subsection *Hexandrae* [46]. These species were grouped together with a bootstrap value of 84% and had the same morphological feature of a hexamerous stemium and a 2n = 14 chromosome number. Notably, *A*. *grandiflora* of subsection *Hexandrae* of the section *Gymnolobus* was transgressively placed into the other clade, while *Aristolochia* sp. was sister to the *Gymnolobus* clade with a very high bootstrap value. Although this unknown taxon could not be identified with any available information, its *rbc*L, *mat*K and ITS2 sequences were unique compared to those of all other *Aristolochia* species ever studied. More investigations of both the chloroplast and nuclear gene sequences of additional *Aristolochia* taxa should be pursued, since these sequences could be used as an efficient molecular identification tool in the future.

## Conclusions

The toxicity of AAs, which are major constituents of *Aristolochia* plant materials, contrasts with the use of traditional knowledge. More studies are critically needed to protect the safety of consumers. The significance of these further studies depends on not only the presence of AAs in samples but also the identities of source plants. Genetic assessment of eleven *Aristolochia* species by DNA barcoding of four standardized DNA regions (*rbc*L, *mat*K, ITS2 and *trn*H-*psb*A) was performed in the present study. DNA barcoding could serve as one effective approach to discriminate among difficult-to-identify plant species of the same genus and expand the global nucleotide database to allow improved detection of plant material identities. The botanical origins of the Thai crude drug called “Krai-Krue” were successfully clarified by a multiplex PCR technique based on the ITS2 region combined with HPTLC profiling. DNA barcoding was used to systematize these materials, along with the chemical profile, and to achieve maximum efficiency for medicinal material identification. These techniques can serve as complementary analytical methods to address the important legal issue of quality control of herbal materials. In the future, natural products should be subjected to the same stringent scrutiny and controls as modern drugs before their release onto the market.

## Supporting information

S1 Fig Sequence alignment of full length *rbc*L genes of eleven *Aristolochia* plants. The numbers on the top line represent the base numbers in sequence alignment. The altered bases indicate the sequence differences. ‘.’ represents the base being identical to the first sequence. The first and the last three nucleotides are start and stop codon, respectively.
(PDF)

S2 Fig Sequence alignment of full length *mat*K genes of eleven *Aristolochia* plants. The numbers on the top line represent the base numbers in sequence alignment. The altered bases indicate the sequence differences. ‘.’ represents the base being identical to the first sequence. The first three nucleotides are start codon and the last three nucleotides are stop codon.
(PDF)

S3 Fig Sequence alignment of *trn*H-*psb*A regions of eleven *Aristolochia* plants. The numbers on the top line represent the base numbers in sequence alignment. The altered bases indicate the sequence differences. ‘.’ represents the base being identical to the first sequence. ‘–’ represents gap.
(PDF)

S4 Fig Sequence alignment of ITS regions of eleven *Aristolochia* plants. The numbers on the top line represent the base numbers in sequence alignment. The altered bases indicate the sequence differences. ‘.’ represents the base being identical to the first sequence. ‘–’ represents gap.
(PDF)

S5 Fig Sequence alignment of ITS2 among eleven *Aristolochia* species and substituted species. Arrows indicate orientation and position of the primer set of multiplex PCR.
(PDF)
