# Supplementary material for: DNA barcoding of Aristolochia plants and development of species-specific multiplex PCR to aid HPTLC in ascertainment of Aristolochia herbal materials
Source: PLoS One. 2018 Aug 20;13(8):e0202625. doi: 10.1371/journal.pone.0202625 (PMC6101415; doi:10.1371/journal.pone.0202625)
Supplement: S1 Fig — The numbers on the top line represent the base numbers in sequence alignment. The altered bases indicate the sequence differences. ‘.’ represents the base being identical to the first sequence. The first and the last three nucleotides are start and stop codon, respectively. (PDF) [file pone.0202625.s001.pdf]

```
                                             1                   2                   3                   4                   5
                           1                 0                   0                   0                   0                   0
A. anguicida (KP903720)    A T G T C A C C A C A A A C A G A G A C T A A A G C A A G T G T T G G A T T C C A A G C T G G T G T
A. gigantea (KP998764)     . . . . . . . . . . . . . . . . . . . . . . . . . . . . . . . . . . . . . . . . . . . . . . . . . .
A. grandiflora (KP998765)  . . . . . . . . . . . . . . . . . . . . . . . . . . . . . . . . . . . . . . . . . . . . . . . . . .
A. kerrii (KP998766)       . . . . . . . . . . . . . . . . . . . . . . . . . . . . . . . . . . . . . . . . . . . . . . . . . .
A. littoralis (KP998767)   . . . . . . . . . . . . . . . . . . . . . . . . . . . . . . . . . . . . . . . . . . . . . . . . . .
A. pierrei (KP998768)      . . . . . . . . . . . . . . . . . . . . . . . . . . . . . . . . . . . . . . . . . . . . . . . . . .
A. pothieri (KP998769)     . . . . . . . . . . . . . . . . . . . . . . . . . . . . . . . . . . . . . . . . . . . . A . . . . .
A. ringens (KP998770)      . . . . . . . . . . . . . . . . . . . . . . . . . . . . . . . . . . . . . . . . . . . . . . . . . .
A. tagala (KP998772)       . . . . . . . . . . . . . . . . . . . . . . . . . . . . . . . . . . . . . . . . . . . . . . . . . .
A. tentaculata (KP998773)  . . . . . . . . . . . . . . . . . . . . . . . . . . . . . . . . . . . . . . . . . . . . . . . . . .
A. sp (KP998771)           . . . . . . . . . . . . . . . . . . . . . . . . . . . . . . . . . . . . . . . . . . . . . . . . . .

                                                                                                                             1
                           5                 6                   7                   8                   9                   0
                           1                 0                   0                   0                   0                   0
A. anguicida (KP903720)    T A A A G A T T A C A A A T T G A C T T A T T A T A C T C C T G A A T A T G A A A C C A A A G A T A
A. gigantea (KP998764)     . . . . . . . . . . . . . . . . . . . . . . . . . . . . . . . . . . . . . . . . . . . . . . . . . .
A. grandiflora (KP998765)  . . . . . . . . . . . . . . . . . . . . . . . . . . . . . . . . . . . . . C . . . . . . . . . . . .
A. kerrii (KP998766)       . . . . . . . . . . . . . . . . . . . . . . . . . . . . . . . . . . . . . C . . . . . . . . . . . .
A. littoralis (KP998767)   . . . . . . . . . . . . . . . . . . . . . . . . . . . . . . . . . . . . . . . . . . . . . . . . . .
A. pierrei (KP998768)      . . . . . . . . . . . . . . . . . . . . . . . . . . . . . . . . . . . . . C . . . . . . . . . . . .
A. pothieri (KP998769)     . . . . . . . . . . . . . . . . . . . . . . . . . . . . . . . . . . . . . C . . . . . . . . . . . .
A. ringens (KP998770)      . . . . . . . . . . . . . . . . . . . . . . . . . . . . . . . . . . . . . . . . . . . . . . . . . .
A. tagala (KP998772)       . . . . . . . . . . . . . . . . . . . . . . . . . . . . . . . . . . . . . C . . . . . . . . . . . .
A. tentaculata (KP998773)  . . . . . . . . . . . . . . . . . . . . . . . . . . . . . . . . . . . . . . . . . . . . . . . . . .
A. sp (KP998771)           . . . . . . . . . . . . . . . . . . . . . . . . . . . . . . . . . . . . . . . . . . . . . . . . . .

                           1                 1                   1                   1                   1                   1
                           0                 1                   2                   3                   4                   5
                           1                 0                   0                   0                   0                   0
A. anguicida (KP903720)    C T G A T A T C T T G G C A G C A T T C C G A G T A A C T C C T C A A C C G G G G G T T C C G C C T
A. gigantea (KP998764)     . . . . . . . . . . . . . . . . . . . . . . . . . . . . . . . . . . . . . . . . . . . . . . . . . .
A. grandiflora (KP998765)  . . . . . . . . . . . . . . . . . . . . . . . . . . . . . . . . . . . . . . . . . . . . . . . . . .
A. kerrii (KP998766)       . . . . . . . . . . . . . . . . . . . . . . . . . . . . . . . . . . . . . . . . . . . . . . . . . .
A. littoralis (KP998767)   . . . . . . . . . . . . . . . . . . . . . . . . . . . . . . . . . . . . . . . . . . . . . . . . . .
A. pierrei (KP998768)      . . . . . . . . . . . . . . . . . . . . . . . . . . . . . . . . . . . . . . . . . . . . . . . . . .
A. pothieri (KP998769)     . . . . . . . . . . . . . . . . . . . . . . . . . . . . . . . . . . . . . . . . . . . . . . . . . .
A. ringens (KP998770)      . . . . . . . . . . . . . . . . . . . . . . . . . . . . . . . . . . . . . . . . . . . . . . . . . .
A. tagala (KP998772)       . . . . . . . . . . . . . . . . . . . . . . . . . . . . . . . . . . . . . . . . . . . . . . . . . .
A. tentaculata (KP998773)  . . . . . . . . . . . . . . . . . . . . . . . . . . . . . . . . . . . . . . . . . . . . . . . . . .
A. sp (KP998771)           . . . . . . . . . . . . . . . . . . . . . . . . . . . . . . . . . . . . . . . . . . . . . . . . . .

                           1                 1                   1                   1                   1                   2
                           5                 6                   7                   8                   9                   0
                           1                 0                   0                   0                   0                   0
A. anguicida (KP903720)    G A G G A A G C A G G G G C T G C A G T A G C T G C C G A A T C T T C T A C T G G T A C A T G G A C
A. gigantea (KP998764)     . . . . . . . . . . . . . . . . . . . . . . . . . . . . . . . . . . . . . . . . . . . . . . . . . .
A. grandiflora (KP998765)  . . . . . . . . . . . . . . . . . . . . . . . . . . . . . . . . . . . . . . A . . . . . . . . . . .
A. kerrii (KP998766)       . . . . . . . . . . . . . . . . . . . . . . . . . . . . . . . . . . . . . . . . . . . . . . . . . .
A. littoralis (KP998767)   . . . . . . . . . . . . . . . . . . . . . . . . . . . . . . . . . . . . . . C . . . . . . . . . . .
A. pierrei (KP998768)      . . . . . . . . . . . . . . . . . . . . . . . . . . . . . . . . . . . . . . . . . . . . . . . . . .
A. pothieri (KP998769)     . . . . . . . . . . . T . . . . . . . . . . . . . . . . . . . . . . . . . . . . . . . . . . . . . .
A. ringens (KP998770)      . . . . . . . . . . . . . . . . . . . . . . . . . . . . . . . . . . . . . . . . . . . . . . . . . .
A. tagala (KP998772)       . . . . . . . . . . . . . . . . . . . . . . . . . . . . . . . . . . . . . . . . . . . . . . . . . .
A. tentaculata (KP998773)  . . . . . . . . . . . . . . . . . . . . . . . . . . . . . . . . . . . G . . . . . . . . . . . . . .
A. sp (KP998771)           . . . . . . . . . . . . . . . . . . . . . . . . . . . . . . . . . . . . . . . . . . . . . . . . . .

                           2                 2                   2                   2                   2                   2
                           0                 1                   2                   3                   4                   5
                           1                 0                   0                   0                   0                   0
A. anguicida (KP903720)    A A C T G T G T G G A C T G A T G G A C T T A C T A G C C T T G A T C G T T A C A A A G G A C G A T
A. gigantea (KP998764)     . . . . . . . . . . . . . . . . . . . . . . . . . . . . . . . . . . . . . . . . . . . . . . . . . .
A. grandiflora (KP998765)  . . . . . . . . . . . . . . . . . . . . . . . . . . . . . . . . . . . . . . . . . . . . . . . . . .
A. kerrii (KP998766)       . . . . . . . . . . . . . . . . . . . . . . . . . . . . . . . . . . . . . . . . . . . . . . . . . .
A. littoralis (KP998767)   . . . . . . . . . . . . . . . . . . . . . . . . . . . . . . . . . . . . . . . . . . . . . . . . . .
A. pierrei (KP998768)      . . . . . . . . . . . . . . . . . . . . . . . . . . . . . . . . . . . . . . . . . . . . . . . . . .
A. pothieri (KP998769)     . . . . . . A . . . . . . . . . . . . . . . . . . . . . . . . . . . . . . . . . . . . . . . . . . .
A. ringens (KP998770)      . . . . . . . . . . . . . . . . . . . . . . . . . . . . . . . . . . . . . . . . . . . . . . . . . .
A. tagala (KP998772)       . . . . . . . . . . . . . . . . . . . . . . . . . . . . . . . . . . . . . . . . . . . . . . . . . .
A. tentaculata (KP998773)  . . . . . . . . . . . . . . . . . . . . . . . . . . . . . . . . . . . . . . . . . . . . . . . . . .
A. sp (KP998771)           . . . . . . . . . . . . . . . . . . . . . . . . . . . . . . . . . . . . . . . . . . . . . . . . . .
```

	251 … 300
A. anguicida (KP903720)	GCTACCACATCGAGCCCGTTGCTGGGGAGGAAAATCAATATATTGCTTAT
A. gigantea (KP998764)	....
A. grandiflora (KP998765)	.....G..
A. kerrii (KP998766)	....................C............G.....T..........
A. littoralis (KP998767)	....
A. pierrei (KP998768)	....................C............G.....T..........
A. pothieri (KP998769)	....................C............G.....T..........
A. ringens (KP998770)	....
A. tagala (KP998772)	....................C............G.....T..........
A. tentaculata (KP998773)	....
A. sp (KP998771)	....

	301 … 350
A. anguicida (KP903720)	GTAGCTTACCCTTTAGACCTTTTTGAAGAAGGTTCTGTTACTAACATGTT
A. gigantea (KP998764)	....
A. grandiflora (KP998765)	....
A. kerrii (KP998766)	....
A. littoralis (KP998767)	....
A. pierrei (KP998768)	....
A. pothieri (KP998769)	....
A. ringens (KP998770)	....
A. tagala (KP998772)	....
A. tentaculata (KP998773)	....
A. sp (KP998771)	....

	351 … 400
A. anguicida (KP903720)	TACTTCCATTGTAGGTAATGTATTTGGGTTCAAAGCCTTACGAGCTTTAC
A. gigantea (KP998764)	....
A. grandiflora (KP998765)	....
A. kerrii (KP998766)	.....................G............................
A. littoralis (KP998767)	....
A. pierrei (KP998768)	.....................G............................
A. pothieri (KP998769)	.....................G............................
A. ringens (KP998770)	....
A. tagala (KP998772)	.....................G............................
A. tentaculata (KP998773)	....
A. sp (KP998771)	....

	401 … 450
A. anguicida (KP903720)	GTCTGGAGGATCTACGAATTCCTCCCGCTTATATCAAAACTTTCCAAGGT
A. gigantea (KP998764)	....
A. grandiflora (KP998765)	.......................A.........................G
A. kerrii (KP998766)	.......................A..T.......................C
A. littoralis (KP998767)	....
A. pierrei (KP998768)	............................G....................C
A. pothieri (KP998769)	.......................A.........................C
A. ringens (KP998770)	....
A. tagala (KP998772)	.......................A....G....................C
A. tentaculata (KP998773)	.......................A.........................C
A. sp (KP998771)	....

	451 … 500
A. anguicida (KP903720)	CCTCCCCATGGTATCCAAGTTGAGAGAGATAAATTGAACAAGTATGGTCG
A. gigantea (KP998764)	....
A. grandiflora (KP998765)	....
A. kerrii (KP998766)	........C...
A. littoralis (KP998767)	....
A. pierrei (KP998768)	........C...
A. pothieri (KP998769)	........C...
A. ringens (KP998770)	....
A. tagala (KP998772)	........C...
A. tentaculata (KP998773)	........C...
A. sp (KP998771)	....

	501–550
A. anguicida (KP903720)	TCCTCTATTGGGATGTACTATTAAACCAAAATTGGGGTTATCGGCGAAGA
A. gigantea (KP998764)	....
A. grandiflora (KP998765)	....
A. kerrii (KP998766)	....
A. littoralis (KP998767)	....
A. pierrei (KP998768)	....
A. pothieri (KP998769)	....
A. ringens (KP998770)	...........................T......................
A. tagala (KP998772)	....
A. tentaculata (KP998773)	....
A. sp (KP998771)	....

	551–600
A. anguicida (KP903720)	ACTACGGTAGAGCGGTTTATGAATGTCTCCGCGGTGGACTTGATTTTACC
A. gigantea (KP998764)	....
A. grandiflora (KP998765)	....
A. kerrii (KP998766)	....
A. littoralis (KP998767)	....
A. pierrei (KP998768)	....
A. pothieri (KP998769)	....
A. ringens (KP998770)	....
A. tagala (KP998772)	....
A. tentaculata (KP998773)	....
A. sp (KP998771)	....

	601–650
A. anguicida (KP903720)	AAGGACGATGAGAACGTGAACTCCCAACCATTTATGCGTTGGAGAGACCG
A. gigantea (KP998764)	....
A. grandiflora (KP998765)	....
A. kerrii (KP998766)	....
A. littoralis (KP998767)	....
A. pierrei (KP998768)	.................A................................
A. pothieri (KP998769)	.................A.............................T..
A. ringens (KP998770)	....
A. tagala (KP998772)	.................A................................
A. tentaculata (KP998773)	....
A. sp (KP998771)	....

	651–700
A. anguicida (KP903720)	TTTCTTATTTTGTGCCGAAGCACTTTATAAAGCACAGGCCGAAACAGGTG
A. gigantea (KP998764)	..............................G...................
A. grandiflora (KP998765)	......................A...........................
A. kerrii (KP998766)	......................A........T..................
A. littoralis (KP998767)	....
A. pierrei (KP998768)	....
A. pothieri (KP998769)	......................A...........................
A. ringens (KP998770)	....
A. tagala (KP998772)	......................A...........................
A. tentaculata (KP998773)	......................A...........................
A. sp (KP998771)	....

	701–750
A. anguicida (KP903720)	AAATCAAAGGACATTACTTAAATGCAACTGCGGGTACATGTGAAGAAATG
A. gigantea (KP998764)	....
A. grandiflora (KP998765)	....
A. kerrii (KP998766)	....
A. littoralis (KP998767)	....
A. pierrei (KP998768)	....
A. pothieri (KP998769)	....
A. ringens (KP998770)	....
A. tagala (KP998772)	....
A. tentaculata (KP998773)	....
A. sp (KP998771)	....

```
                            7        7         7         7         7         8
                            5        6         7         8         9         0
                            1        0         0         0         0         0
A. anguicida (KP903720)     ATGAAAAGGGCGATATTTGCCAGAGAATTGGGAGTTCCTATCGTTATGCA
A. gigantea (KP998764)      ..................................................
A. grandiflora (KP998765)   ...........C......................C...............
A. kerrii (KP998766)        ...........C......................................
A. littoralis (KP998767)    ..................................................
A. pierrei (KP998768)       ...........C........T.............................
A. pothieri (KP998769)      ...........C....G...T.............................
A. ringens (KP998770)       ..................................................
A. tagala (KP998772)        ...........C........T.............................
A. tentaculata (KP998773)   ...........T....G.................................
A. sp (KP998771)            ..................................................

                            8        8         8         8         8         8
                            0        1         2         3         4         5
                            1        0         0         0         0         0
A. anguicida (KP903720)     TGACTACTTAACGGGAGGATTCACTGCAAATACTAGCTTGGCTCATTATT
A. gigantea (KP998764)      ..................................................
A. grandiflora (KP998765)   ..................................................
A. kerrii (KP998766)        ..................................................
A. littoralis (KP998767)    ..................................................
A. pierrei (KP998768)       ..................................................
A. pothieri (KP998769)      ..................................................
A. ringens (KP998770)       ..................................................
A. tagala (KP998772)        ..................................................
A. tentaculata (KP998773)   ..................................................
A. sp (KP998771)            ..................................................

                            8        8         8         8         8         9
                            5        6         7         8         9         0
                            1        0         0         0         0         0
A. anguicida (KP903720)     GCCGAGACAATGGCCTACTTCTTCACATCCATCGCGCAATGCATGCAGTT
A. gigantea (KP998764)      ..................................................
A. grandiflora (KP998765)   ...............................C..................
A. kerrii (KP998766)        .............A....................................
A. littoralis (KP998767)    ..................................................
A. pierrei (KP998768)       ..................................................
A. pothieri (KP998769)      ...............................C..................
A. ringens (KP998770)       ..................................................
A. tagala (KP998772)        ..................................................
A. tentaculata (KP998773)   ..................................................
A. sp (KP998771)            ..................................................

                            9        9         9         9         9         9
                            0        1         2         3         4         5
                            1        0         0         0         0         0
A. anguicida (KP903720)     ATTGATAGACAGAAGAATCATGGTATGCACTTCCGAGTACTTGCAAAAGC
A. gigantea (KP998764)      ..................................................
A. grandiflora (KP998765)   ..................................................
A. kerrii (KP998766)        ...........A......................................
A. littoralis (KP998767)    ..................................................
A. pierrei (KP998768)       ..................................................
A. pothieri (KP998769)      ..................................................
A. ringens (KP998770)       ..................................................
A. tagala (KP998772)        ..................................................
A. tentaculata (KP998773)   ..................................................
A. sp (KP998771)            ..................................................

                                                                             1
                            9        9         9         9         9         0
                            5        6         7         8         9         0
                            1        0         0         0         0         0
A. anguicida (KP903720)     ATTACGTTTGTCCGGTGGAGATCATATTCACGCCGGTACCGTAGTAGGTA
A. gigantea (KP998764)      ..................................................
A. grandiflora (KP998765)   ..................................................
A. kerrii (KP998766)        ..................................................
A. littoralis (KP998767)    ..................................................
A. pierrei (KP998768)       .......A..........................................
A. pothieri (KP998769)      .......A..........................................
A. ringens (KP998770)       ..................................................
A. tagala (KP998772)        .......A..........................................
A. tentaculata (KP998773)   ..................................................
A. sp (KP998771)            ..................................................
```

```
                           1        1         1         1         1         1
                           0        0         0         0         0         0
                           0        1         2         3         4         5
                           1        0         0         0         0         0
A. anguicida (KP903720)    AACTGGAAGGGGAACGGGAAATCACTTTGGGTTTTGTTGATTTACTACGT
A. gigantea (KP998764)     ..................................................
A. grandiflora (KP998765)  ..................................................
A. kerrii (KP998766)       ..................................................
A. littoralis (KP998767)   ..................................................
A. pierrei (KP998768)      ..........T.......................................
A. pothieri (KP998769)     ..........T.......................................
A. ringens (KP998770)      ..................................................
A. tagala (KP998772)       ..........T.......................................
A. tentaculata (KP998773)  ..................................................
A. sp (KP998771)           ..................................................

                           1        1         1         1         1         1
                           0        0         0         0         0         1
                           5        6         7         8         9         0
                           1        0         0         0         0         0
A. anguicida (KP903720)    GATGATTTTGTTGAAAAAGACCGAAGTCGCGGTATTTATTTCACTCAAGA
A. gigantea (KP998764)     .......A..........................................
A. grandiflora (KP998765)  .......A..........................................
A. kerrii (KP998766)       .......A..........................................
A. littoralis (KP998767)   .......A..........................................
A. pierrei (KP998768)      .......A..........................................
A. pothieri (KP998769)     .......A..........................................
A. ringens (KP998770)      .......A..........................................
A. tagala (KP998772)       .......A..........................................
A. tentaculata (KP998773)  .......A..........................................
A. sp (KP998771)           ..................................................

                           1        1         1         1         1         1
                           1        1         1         1         1         1
                           0        1         2         3         4         5
                           1        0         0         0         0         0
A. anguicida (KP903720)    TTGGGTCTCTCTTCCGGGTGTTCTGCCCGTGGCTTCCGGAGGTATTCACG
A. gigantea (KP998764)     ..................................................
A. grandiflora (KP998765)  ..................................................
A. kerrii (KP998766)       ...............A......T...........................
A. littoralis (KP998767)   ..................................................
A. pierrei (KP998768)      ..................................................
A. pothieri (KP998769)     ..................................................
A. ringens (KP998770)      ..................................................
A. tagala (KP998772)       ..................................................
A. tentaculata (KP998773)  ..................................................
A. sp (KP998771)           ..................................................

                           1        1         1         1         1         1
                           1        1         1         1         1         2
                           5        6         7         8         9         0
                           1        0         0         0         0         0
A. anguicida (KP903720)    TTTGGCATATGCCTGCCCTGACCGAGATCTTTGGGGATGATTCCGTACTA
A. gigantea (KP998764)     ..................................................
A. grandiflora (KP998765)  ..................................................
A. kerrii (KP998766)       ..................................................
A. littoralis (KP998767)   ..................................................
A. pierrei (KP998768)      ..................................................
A. pothieri (KP998769)     ..................................................
A. ringens (KP998770)      ..................................................
A. tagala (KP998772)       ..................................................
A. tentaculata (KP998773)  ..................................................
A. sp (KP998771)           ..................................................

                           1        1         1         1         1         1
                           2        2         2         2         2         2
                           0        1         2         3         4         5
                           1        0         0         0         0         0
A. anguicida (KP903720)    CAGTTCGGCGGAGGAACTTTAGGACACCCTTGGGGAAATGCACCTGGTGC
A. gigantea (KP998764)     ..................................................
A. grandiflora (KP998765)  .....T............................................
A. kerrii (KP998766)       ........T.........................................
A. littoralis (KP998767)   ..................................................
A. pierrei (KP998768)      ..................................................
A. pothieri (KP998769)     ..................................................
A. ringens (KP998770)      ..................................................
A. tagala (KP998772)       ..................................................
A. tentaculata (KP998773)  ............................................A....G
A. sp (KP998771)           ..................................................
```

Species	1251–1300
A. anguicida (KP903720)	AGTAGCTAATAGGGTGGCTTTAGAAGCGTGTGTACAAGCTCGTAACGAGG
A. gigantea (KP998764)	....
A. grandiflora (KP998765)	....T....
A. kerrii (KP998766)	....T....
A. littoralis (KP998767)	....
A. pierrei (KP998768)	....T....
A. pothieri (KP998769)	....T....
A. ringens (KP998770)	....
A. tagala (KP998772)	....T....
A. tentaculata (KP998773)	....T....
A. sp (KP998771)	....

Species	1301–1350
A. anguicida (KP903720)	GGCGTGATCTTGCTCGCGAAGGTAATGAAGTTATCCGTGAAGCTGCCAAA
A. gigantea (KP998764)	....
A. grandiflora (KP998765)	................T..........C......................
A. kerrii (KP998766)	................T............A....................
A. littoralis (KP998767)	....
A. pierrei (KP998768)	................T.................................
A. pothieri (KP998769)	................T.................................
A. ringens (KP998770)	................T.................................
A. tagala (KP998772)	................T.................................
A. tentaculata (KP998773)	................T.................................
A. sp (KP998771)	....

Species	1351–1400
A. anguicida (KP903720)	TGGAGCCCTGAACTAGCGGCTGCTTGTGAAGTATGGAAGGAGATCAAATT
A. gigantea (KP998764)	....
A. grandiflora (KP998765)	....
A. kerrii (KP998766)	....T.....
A. littoralis (KP998767)	....
A. pierrei (KP998768)	..............T...................................
A. pothieri (KP998769)	..............T...................................
A. ringens (KP998770)	....
A. tagala (KP998772)	..............T...................................
A. tentaculata (KP998773)	....
A. sp (KP998771)	....

Species	1401–1428
A. anguicida (KP903720)	CGAATTCGAAGCAATGGATACCTTGTAA
A. gigantea (KP998764)	....
A. grandiflora (KP998765)	.......CCG..................
A. kerrii (KP998766)	.......CC...................
A. littoralis (KP998767)	....
A. pierrei (KP998768)	.......CCG..................
A. pothieri (KP998769)	.......CCG..................
A. ringens (KP998770)	....
A. tagala (KP998772)	.......CCG..................
A. tentaculata (KP998773)	.......CCG..................
A. sp (KP998771)	....
