# Supplementary material for: DNA barcoding of Aristolochia plants and development of species-specific multiplex PCR to aid HPTLC in ascertainment of Aristolochia herbal materials
Source: PLoS One. 2018 Aug 20;13(8):e0202625. doi: 10.1371/journal.pone.0202625 (PMC6101415; doi:10.1371/journal.pone.0202625)
Supplement: S2 Fig — The numbers on the top line represent the base numbers in sequence alignment. The altered bases indicate the sequence differences. ‘.’ represents the base being identical to the first sequence. The first three nucleotides are start codon and the last three nucleotides are stop codon. (PDF) [file pone.0202625.s002.pdf]

```
                           1        10        20        30        40        50
A. anguicida (KP998777)    ATGGAGGAATTACAAGGATATTTAGAAATGGGTAGATCTCCACAAAAACA
A. gigantea (KP998778)     ...............................A..................
A. grandiflora (KP998779)  ..................................................
A. kerrii (KP998780)       ..................................................
A. littoralis(KP998781)    ...............................A..................
A. pierrei (KP998782)      ..................................................
A. pothieri (KP998783)     ....G............................G.......G........
A. ringens (KP998784)      .........................................G........
A. tagala (KP998786)       ..................................................
A. tentaculata (KP998787)  ...............................T..................
A. sp (KP998785)           ....G............................G.......G........

                           51       60        70        80        90        100
A. anguicida (KP998777)    CTTCCTATATCCACTTCTCTTTCAGGAGTCTATCTATGTACTTGCTCATA
A. gigantea (KP998778)     ..................................................
A. grandiflora (KP998779)  ..........A.......................................
A. kerrii (KP998780)       ................................C.................
A. littoralis(KP998781)    ..................................................
A. pierrei (KP998782)      ..................................................
A. pothieri (KP998783)     ......................................C...........
A. ringens (KP998784)      ..................................................
A. tagala (KP998786)       ..................................................
A. tentaculata (KP998787)  ..................................................
A. sp (KP998785)           ......................................C...........

                           101      110       120       130       140       150
A. anguicida (KP998777)    ATCATGATTTAAATGGATCAATTGTTTACGAATCGATGGAAAATTTAGGT
A. gigantea (KP998778)     ..................................................
A. grandiflora (KP998779)  ...................G..............................
A. kerrii (KP998780)       ..T.......T........G..............................
A. littoralis(KP998781)    ..................................................
A. pierrei (KP998782)      ...................G..............................
A. pothieri (KP998783)     ...................G...................G..........
A. ringens (KP998784)      ..................................................
A. tagala (KP998786)       ...................G..............................
A. tentaculata (KP998787)  ...................G..............................
A. sp (KP998785)           ...................G...................G..........

                           151      160       170       180       190       200
A. anguicida (KP998777)    TATGACAAAAGATTCAGTTCACTAATTGTGAAACGTTTAATTATTCGAAT
A. gigantea (KP998778)     ..................................................
A. grandiflora (KP998779)  ..........A...A...................................
A. kerrii (KP998780)       ..........A............T..........................
A. littoralis(KP998781)    ..................................................
A. pierrei (KP998782)      ..........A.......................................
A. pothieri (KP998783)     ..........A...A....T..............................
A. ringens (KP998784)      .....................A............................
A. tagala (KP998786)       ..........A.......................................
A. tentaculata (KP998787)  ..................................................
A. sp (KP998785)           ..........A...A....T..............................

                           201      210       220       230       240       250
A. anguicida (KP998777)    GCATCAACAGAACCATTTTTTTATTTTGGTTACCAATTTT------CACC
A. gigantea (KP998778)     ...................A....................------A...
A. grandiflora (KP998779)  .............T............C.......T.....------....
A. kerrii (KP998780)       ..............C....A......C.......T.....------....
A. littoralis(KP998781)    ...................A....................------....
A. pierrei (KP998782)      ..............------..T...C.............------....
A. pothieri (KP998783)     ..............------..T...C.............CACAAA...A
A. ringens (KP998784)      ..........................C.............------....
A. tagala (KP998786)       ..............------..T...C.............------....
A. tentaculata (KP998787)  ..........................C.............------....
A. sp (KP998785)           ..............------..T...C.............CACAAA...A
```

	251 260 270 280 290 300
A. anguicida (KP998777)	AAAATAGATTCGTTAGGAAAAGCAATAATTTGGATTATCAAACCATATCA
A. gigantea (KP998778)	.....G..
A. grandiflora (KP998779)	..............G......CA..A......T...C.............
A. kerrii (KP998780)	.....G.........AA.........T....T....C.............
A. littoralis(KP998781)	....
A. pierrei (KP998782)	......................A..A...G......C.............
A. pothieri (KP998783)	....GG................A..A...G......C.....T.......
A. ringens (KP998784)	....................................C.............
A. tagala (KP998786)	......................A..A...G......C.............
A. tentaculata (KP998787)	....................................C.............
A. sp (KP998785)	....GG................A..A...G......C.....T.......

	301 310 320 330 340 350
A. anguicida (KP998777)	GAGGCTTTTGCAGTCATTATGGAAATTCCACTCTCCCTGCAATTAGTATC
A. gigantea (KP998778)	....
A. grandiflora (KP998779)	....
A. kerrii (KP998780)	....
A. littoralis(KP998781)	....
A. pierrei (KP998782)	....
A. pothieri (KP998783)	..............T......................C............
A. ringens (KP998784)	....
A. tagala (KP998786)	....
A. tentaculata (KP998787)	....................................AG............
A. sp (KP998785)	..............T......................C............

	351 360 370 380 390 400
A. anguicida (KP998777)	TTGCCTGGAAGAAAAAGAAATAGCCAAATCTCATAATTTACGATCTATTC
A. gigantea (KP998778)	........................A.........................
A. grandiflora (KP998779)	.....A..................A.........................
A. kerrii (KP998780)	.............C..........A.........................
A. littoralis(KP998781)	........................A.........................
A. pierrei (KP998782)	........................A....A....................
A. pothieri (KP998783)	........................A....A...A................
A. ringens (KP998784)	........................A.........................
A. tagala (KP998786)	........................A....A....................
A. tentaculata (KP998787)	........................A.........................
A. sp (KP998785)	........................A....A...A................

	401 410 420 430 440 450
A. anguicida (KP998777)	ATTCAATATTTCCCTTTTTCGAGGACAAATTATCACATTTAAATCATGTG
A. gigantea (KP998778)	....
A. grandiflora (KP998779)	..........C.......................................
A. kerrii (KP998780)	...................T..............................
A. littoralis(KP998781)	....
A. pierrei (KP998782)	...................T..............................
A. pothieri (KP998783)	...................T..............................
A. ringens (KP998784)	....
A. tagala (KP998786)	...................T..............................
A. tentaculata (KP998787)	....
A. sp (KP998785)	...................T..............................

	451 460 470 480 490 500
A. anguicida (KP998777)	TTAGATATCCTAATACCCCACCCCGTCCATCTGGAAATCTTGGTTCAAAC
A. gigantea (KP998778)	....
A. grandiflora (KP998779)	........................A.........................
A. kerrii (KP998780)	........................A.........................
A. littoralis(KP998781)	....
A. pierrei (KP998782)	........................A.........................
A. pothieri (KP998783)	.................T......A.........................
A. ringens (KP998784)	.........................C........................
A. tagala (KP998786)	........................A.........................
A. tentaculata (KP998787)	....
A. sp (KP998785)	.................T......A.........................

```
Positions 501–550
A. anguicida (KP998777)      CCTTCACTCCTGGATACAAGATGCTCCCTCTTTGCATTTATTGAGATTTT
A. gigantea (KP998778)       ..................................................
A. grandiflora (KP998779)    ........G.........................................
A. kerrii (KP998780)         ........G..............T.....T....................
A. littoralis(KP998781)      ..................................................
A. pierrei (KP998782)        ........G........................A................
A. pothieri (KP998783)       ........G.......A......T.........A................
A. ringens (KP998784)        ..................................................
A. tagala (KP998786)         ........G........................A................
A. tentaculata (KP998787)    ..................................................
A. sp (KP998785)             ........G.......A.................................

Positions 551–600
A. anguicida (KP998777)      TTCTCCACGAGTATCGTAATTCAAATAGTTTCATTAATTCAAAGAGATCC
A. gigantea (KP998778)       ..................................................
A. grandiflora (KP998779)    ............T..A.............................A....
A. kerrii (KP998780)         ....................................G........A....
A. littoralis(KP998781)      ...............A..................................
A. pierrei (KP998782)        ...............C....................G........A....
A. pothieri (KP998783)       ...............C....................G......A.A....
A. ringens (KP998784)        .............................................A...T
A. tagala (KP998786)         ...............C....................G........A....
A. tentaculata (KP998787)    .......G.....................................A...T
A. sp (KP998785)             ..................................................

Positions 601–650
A. anguicida (KP998777)      TTTTTTTTTTTCTC---------------AAAAGAGAATCAAAGAAAAGA
A. gigantea (KP998778)       ..............AAAAGAGAATCAAAG.....................
A. grandiflora (KP998779)    A.....A....A..------------------------------......
A. kerrii (KP998780)         ...........T..---------------------------TCA...GA.
A. littoralis(KP998781)      ..............---------------.....................
A. pierrei (KP998782)        A...C.........------------------------------......
A. pothieri (KP998783)       A...C.........------------------------------.....G
A. ringens (KP998784)        A.............------------------------------......
A. tagala (KP998786)         A...C.........------------------------------......
A. tentaculata (KP998787)    A.............------------------------------......
A. sp (KP998785)             ..............---------------.....................

Positions 651–700
A. anguicida (KP998777)      GAATCAAAGATTATTCTTGTTCCTATATAATTCTCATGTATATGAATGCG
A. gigantea (KP998778)       ..................................................
A. grandiflora (KP998779)    .................................G................
A. kerrii (KP998780)         ............C........T............................
A. littoralis(KP998781)      .................................................A
A. pierrei (KP998782)        ..................................................
A. pothieri (KP998783)       ..................................................
A. ringens (KP998784)        ..................................................
A. tagala (KP998786)         ..................................................
A. tentaculata (KP998787)    ...............A..................................
A. sp (KP998785)             ..................................................

Positions 701–750
A. anguicida (KP998777)      AATCCATATTCGTTTTTCTCGGTAAACAAGCCTCTCGTTTACGATCAACA
A. gigantea (KP998778)       ..................................................
A. grandiflora (KP998779)    ..........GT......................................
A. kerrii (KP998780)         ..................................................
A. littoralis(KP998781)      ..........................................A.......
A. pierrei (KP998782)        .....C........................T...................
A. pothieri (KP998783)       ..........T...................T...................
A. ringens (KP998784)        ..................................................
A. tagala (KP998786)         ..............................T...................
A. tentaculata (KP998787)    ....................................A.............
A. sp (KP998785)             ..................................................
```

	751 760 770 780 790 800
A. anguicida (KP998777)	TCCCCTGGAGCCTTTCTTGAGCGAACATATTTCTATGGAAAAATGGAGCA
A. gigantea (KP998778)	....
A. grandiflora (KP998779)	.....................................T.......A....
A. kerrii (KP998780)	............C...............T.....................
A. littoralis(KP998781)	....
A. pierrei (KP998782)	....T...
A. pothieri (KP998783)	....T...
A. ringens (KP998784)	....
A. tagala (KP998786)	....T...
A. tentaculata (KP998787)	....
A. sp (KP998785)	....

	801 810 820 830 840 850
A. anguicida (KP998777)	TTTTTTAGTAGTGCTTTGTAATCATTTTCAGAAGACCTTGTGGTTACTTA
A. gigantea (KP998778)	..............................A...................
A. grandiflora (KP998779)	.....................................C............
A. kerrii (KP998780)	.....................................C............
A. littoralis(KP998781)	...........G..................A..A......G.........
A. pierrei (KP998782)	.....................................C............
A. pothieri (KP998783)	.....................................C............
A. ringens (KP998784)	....
A. tagala (KP998786)	.....................................C............
A. tentaculata (KP998787)	......................T...........................
A. sp (KP998785)	..............................A..A......G.........

	851 860 870 880 890 900
A. anguicida (KP998777)	AGGATCCTTTTGCACATTATGTCAGATACCGAGGAAAATCAATTCTGGCT
A. gigantea (KP998778)	........................A.........................
A. grandiflora (KP998779)	.............G....................................
A. kerrii (KP998780)	.............G....................................
A. littoralis(KP998781)	........................A.........................
A. pierrei (KP998782)	.............G....................................
A. pothieri (KP998783)	.............G..............................A..T..
A. ringens (KP998784)	....
A. tagala (KP998786)	.............G....................................
A. tentaculata (KP998787)	.............G....................................
A. sp (KP998785)	........................A.........................

	901 910 920 930 940 950
A. anguicida (KP998777)	TCAAAGGGGACTCATTTTATGATGAAGAAATGGAAATATCATCTTGTCAA
A. gigantea (KP998778)	..........................A.......................
A. grandiflora (KP998779)	........T......C.......................T..........
A. kerrii (KP998780)	............T..C.......................T..........
A. littoralis(KP998781)	..........................A.......................
A. pierrei (KP998782)	...............C.......................T..........
A. pothieri (KP998783)	........A......C.......................T..........
A. ringens (KP998784)	....
A. tagala (KP998786)	...............C.......................T..........
A. tentaculata (KP998787)	....
A. sp (KP998785)	..........................A.......................

	951 960 970 980 990 1000
A. anguicida (KP998777)	TTTATGGCAGTGTCATTTTCACTTGTGGTCTCAACCGGGCAGAATCCATA
A. gigantea (KP998778)	.........................G........................
A. grandiflora (KP998779)	.............T....................................
A. kerrii (KP998780)	........................C.........................
A. littoralis(KP998781)	.........................G...............A........
A. pierrei (KP998782)	....T..........
A. pothieri (KP998783)	......................................AT..........
A. ringens (KP998784)	....
A. tagala (KP998786)	....T..........
A. tentaculata (KP998787)	....
A. sp (KP998785)	.........................G........................

	1001 1010 1020 1030 1040 1050
A. anguicida (KP998777)	TAAACCGATTATGCAATCATTCCTTCTATTTTATGGGCTATCTCTCAAGT
A. gigantea (KP998778)	......A...
A. grandiflora (KP998779)	......A.....A.....................................
A. kerrii (KP998780)	...........GA....A...T............................
A. littoralis(KP998781)	....
A. pierrei (KP998782)	............A.....................................
A. pothieri (KP998783)	......A.....A.....................................
A. ringens (KP998784)	............A.....................................
A. tagala (KP998786)	............A.....................................
A. tentaculata (KP998787)	............A..............T......................
A. sp (KP998785)	....

	1051 1060 1070 1080 1090 1100
A. anguicida (KP998777)	CTACGAATGGTCCC---------TTCGATGGTAAGGAGTCAAATGCTAGA
A. gigantea (KP998778)	..............---------...........................
A. grandiflora (KP998779)	.........T....---------.....A................A....
A. kerrii (KP998780)	...........T..---------C..........................
A. littoralis(KP998781)	..............---------...........................
A. pierrei (KP998782)	............G.---------...A.......................
A. pothieri (KP998783)	.......A....G.---------......A....................
A. ringens (KP998784)	..............TTCGATGGT...........................
A. tagala (KP998786)	............G.---------...A.......................
A. tentaculata (KP998787)	..............---------...........................
A. sp (KP998785)	..............---------...........................

	1101 1110 1120 1130 1140 1150
A. anguicida (KP998777)	AAATTCATTTCTAATAGATACTTCTATTAAGAGATTCGAGACCATAGTTC
A. gigantea (KP998778)	....G...
A. grandiflora (KP998779)	....C......
A. kerrii (KP998780)	..........A...CG...........................C......
A. littoralis(KP998781)	....G...
A. pierrei (KP998782)	....
A. pothieri (KP998783)	....G......................................T.G....
A. ringens (KP998784)	....C......
A. tagala (KP998786)	....
A. tentaculata (KP998787)	....G...
A. sp (KP998785)	....

	1151 1160 1170 1180 1190 1200
A. anguicida (KP998777)	CAATTTTTTCTCTAATTAGTTCTTTGGCTAAAGCGAAATTTTGTAATGTA
A. gigantea (KP998778)	.C..
A. grandiflora (KP998779)	.T.......A......G.....C...T.......T...............
A. kerrii (KP998780)	.C..............A.....C...T.......................
A. littoralis(KP998781)	....
A. pierrei (KP998782)	......A.........A.........T.......................
A. pothieri (KP998783)	......A.........A.....C...T...........C...........
A. ringens (KP998784)	....A...
A. tagala (KP998786)	................A.....C...T.......................
A. tentaculata (KP998787)	....
A. sp (KP998785)	....

	1201 1210 1220 1230 1240 1250
A. anguicida (KP998777)	TCAGGACATCCCATTAGTAAGCCGGCTCGGTCTGATTCGTCTGATTTTGA
A. gigantea (KP998778)	....C........
A. grandiflora (KP998779)	.....................T..........G........C........
A. kerrii (KP998780)	...........................T......................
A. littoralis(KP998781)	....C........
A. pierrei (KP998782)	....C........
A. pothieri (KP998783)	........................A................C........
A. ringens (KP998784)	....CA.......
A. tagala (KP998786)	....C........
A. tentaculata (KP998787)	....C........
A. sp (KP998785)	....

	1251 · 1260 · 1270 · 1280 · 1290 · 1300
A. anguicida (KP998777)	TATTATTGATCGATTTGGCCATATATGCAGAAATCTTTCTCATTATTACA
A. gigantea (KP998778)	....
A. grandiflora (KP998779)	......C...
A. kerrii (KP998780)	......C.............G.............A...............
A. littoralis(KP998781)	....
A. pierrei (KP998782)	......C.............G.............A...............
A. pothieri (KP998783)	......C.............G.............A...............
A. ringens (KP998784)	....................G.............................
A. tagala (KP998786)	......C.............G.............A...............
A. tentaculata (KP998787)	......C.............G.............................
A. sp (KP998785)	....

	1301 · 1310 · 1320 · 1330 · 1340 · 1350
A. anguicida (KP998777)	GTGGATCCTCAAAAAAACATAGTTTGTATCGAATCAAGTATATACTTCGA
A. gigantea (KP998778)	....
A. grandiflora (KP998779)	....
A. kerrii (KP998780)	....
A. littoralis(KP998781)	....
A. pierrei (KP998782)	....
A. pothieri (KP998783)	.................A.G..............................
A. ringens (KP998784)	....
A. tagala (KP998786)	....
A. tentaculata (KP998787)	....
A. sp (KP998785)	....

	1351 · 1360 · 1370 · 1380 · 1390 · 1400
A. anguicida (KP998777)	CTTTCATGTGCTAGAACTTTGGCTCGTAAACACAAAAGTACGGTACGCAC
A. gigantea (KP998778)	....
A. grandiflora (KP998779)	....
A. kerrii (KP998780)	....
A. littoralis(KP998781)	....
A. pierrei (KP998782)	....
A. pothieri (KP998783)	....
A. ringens (KP998784)	....
A. tagala (KP998786)	....
A. tentaculata (KP998787)	....
A. sp (KP998785)	....

	1401 · 1410 · 1420 · 1430 · 1440 · 1450
A. anguicida (KP998777)	CTTTTTGAAAAAATTAGGTTCGGAATTCTTAGAGGAATTCCTTATGGAGG
A. gigantea (KP998778)	....
A. grandiflora (KP998779)	T.....T.....................................C.....
A. kerrii (KP998780)	T...C...A.
A. littoralis(KP998781)	............T.....................................
A. pierrei (KP998782)	T.......................................T...C...A.
A. pothieri (KP998783)	T..........................A............T...C...A.
A. ringens (KP998784)	....C.....
A. tagala (KP998786)	T.......................................T...C...A.
A. tentaculata (KP998787)	....C.....
A. sp (KP998785)	....

	1451 · 1460 · 1470 · 1480 · 1490 · 1500
A. anguicida (KP998777)	AAGAACAAGTTCTTTCTTTGATCTTCCCAAGAACCTCTTCTATTTCACAT
A. gigantea (KP998778)	....
A. grandiflora (KP998779)	...........A..................................G...
A. kerrii (KP998780)	....G...
A. littoralis(KP998781)	....
A. pierrei (KP998782)	....G...
A. pothieri (KP998783)	....A.....G...
A. ringens (KP998784)	...........T..................................G...
A. tagala (KP998786)	....G...
A. tentaculata (KP998787)	.....................................C........G...
A. sp (KP998785)	....

	1501									1510										1520										1530										1540										1550
A. anguicida **(KP998777)**	A	A	G	T	T	A	C	A	T	A	A	G	G	A	A	T	G	T	A	T	T	T	G	G	T	A	T	T	T	G	G	A	T	A	T	T	A	T	C	C	G	T	A	T	C	A	C	C	G	A
A. gigantea **(KP998778)**	.	.	.	.	.	.	.	.	.	.	.	.	.	.	.	.	.	.	.	.	.	.	.	.	.	.	.	.	.	.	.	.	.	.	.	.	.	.	.	.	.	.	.	.	.	.	.	.	.	.
A. grandiflora **(KP998779)**	.	G	.	.	.	.	.	.	.	.	.	.	.	.	.	.	.	.	.	.	.	.	.	.	.	.	.	.	.	.	.	.	.	.	.	.	T	.	.	.	.	.	.	.	.	.	.	T	.	.
A. kerrii **(KP998780)**	.	G	.	.	.	.	.	.	.	.	.	.	.	.	.	.	.	.	.	.	.	.	.	.	.	.	.	.	.	.	.	.	.	.	.	.	.	.	.	.	.	.	.	.	.	.	.	T	.	.
A. littoralis**(KP998781)**	.	.	.	.	.	.	.	.	.	.	.	.	.	.	.	.	.	.	.	.	.	.	.	.	.	.	.	.	.	.	.	.	.	.	.	.	.	.	.	.	.	.	.	.	.	.	.	.	.	.
A. pierrei **(KP998782)**	.	G	.	.	.	.	.	.	.	.	.	.	.	.	.	.	.	.	.	.	.	.	.	.	.	.	.	.	.	.	.	.	.	.	.	.	.	.	.	.	.	.	.	.	.	G	.	T	.	.
A. pothieri **(KP998783)**	.	G	.	.	.	.	.	.	.	.	.	.	.	.	.	.	T	.	C	.	.	.	.	.	.	.	.	.	.	.	.	.	.	.	.	.	.	.	.	.	.	.	.	.	.	.	.	T	.	.
A. ringens **(KP998784)**	.	G	.	.	.	.	.	.	.	.	.	.	.	.	.	.	.	.	.	.	.	.	.	.	.	.	.	.	.	.	.	.	.	.	.	.	.	.	.	.	.	.	.	.	.	.	.	T	.	.
A. tagala **(KP998786)**	.	G	.	.	.	.	.	.	.	.	.	.	.	.	.	.	.	.	.	.	.	.	.	.	.	.	.	.	.	.	.	.	.	.	.	.	.	.	.	.	.	.	.	.	.	G	.	T	.	.
A. tentaculata **(KP998787)**	.	G	.	.	.	.	.	.	.	.	.	.	.	.	.	.	.	.	.	.	.	.	.	.	.	.	.	.	.	.	.	.	.	.	.	.	.	.	.	.	.	.	.	.	.	.	.	T	.	.
A. **sp (KP998785)**	.	.	.	.	.	.	.	.	.	.	.	.	.	.	.	.	.	.	.	.	.	.	.	.	.	.	.	.	.	.	.	.	.	.	.	.	.	.	.	.	.	.	.	.	.	.	.	.	.	.

	1551									1560									1569
A. anguicida **(KP998777)**	C	C	T	G	G	C	T	A	A	T	C	A	T	G	A	A	T	G	A
A. gigantea **(KP998778)**	.	T	.	.	.	.	.	.	.	.	.	.	.	.	.	.	.	.	.
A. grandiflora **(KP998779)**	.	.	.	.	.	.	C	.	.	.	T	.	C	T	C	.	.	.	.
A. kerrii **(KP998780)**	.	.	.	.	.	.	.	.	.	.	.	.	.	T	C	.	.	.	.
A. littoralis**(KP998781)**	.	.	.	.	.	.	.	.	.	.	.	.	.	.	.	.	.	.	.
A. pierrei **(KP998782)**	.	.	.	.	.	.	.	.	.	C	.	.	.	T	C	.	.	.	.
A. pothieri **(KP998783)**	.	.	.	.	.	.	C	.	.	.	.	.	.	T	C	.	.	.	.
A. ringens **(KP998784)**	.	.	.	.	.	.	C	.	.	.	.	.	.	T	C	.	.	.	.
A. tagala **(KP998786)**	.	.	.	.	.	.	.	.	.	.	.	.	.	T	C	.	.	.	.
A. tentaculata **(KP998787)**	.	.	.	.	.	.	C	.	.	.	.	.	.	.	.	.	.	.	.
A. **sp (KP998785)**	.	.	.	.	.	.	.	.	.	.	.	.	.	.	.	.	.	.	.
