# Supplementary material for: DNA barcoding of Aristolochia plants and development of species-specific multiplex PCR to aid HPTLC in ascertainment of Aristolochia herbal materials
Source: PLoS One. 2018 Aug 20;13(8):e0202625. doi: 10.1371/journal.pone.0202625 (PMC6101415; doi:10.1371/journal.pone.0202625)
Supplement: S3 Fig — The numbers on the top line represent the base numbers in sequence alignment. The altered bases indicate the sequence differences. ‘.’ represents the base being identical to the first sequence. ‘–’ represents gap. (PDF) [file pone.0202625.s003.pdf]

```
                                   1         2         3         4         5
                          1        0         0         0         0         0
A. anguicida (KP998805)   --------------------------------TCTAGACCTGGCTGCTCT
A. gigantea (KP998806)    -----------------------CAACTTCCC..................
A. grandiflora (KP998807) GGTATGCATGAACGTAATGCTCACAACTTCCC..................
A. kerrii (KP998808)      GGTATGCATGAACGTAATGCTCACAACTTCCC..................
A. littoralis (KP998809)  ------------------GCTCACAACTTCCC..................
A. pierrei (KP998810)     ---------------AATGCTCACAACTTCCC..................
A. pothieri (KP998811)    GGTATGCATGAACGTAATGCTCACAACTTCCC..................
A. ringens (KP998812)     -----------------------CAACTTCCC..................
A. tagala (KP998814)      -----------------TGCTCACAACTTCCC..................
A. tentaculata (KP998815) --------TGAACGTAATGCTCACAACTTCCC......T...........
A. sp (KP998813)          -----------------TGCTCACAACTTCCC..................

                                                                           1
                          5        6         7         8         9         0
                          1        0         0         0         0         0
A. anguicida (KP998805)   TGAAGCTCCATCTACAAATGGATAATACATCGGT-----ATTAGTGTATA
A. gigantea (KP998806)    ..................................-----...........
A. grandiflora (KP998807) ...............G..................-----...........
A. kerrii (KP998808)      ..................................-----...........
A. littoralis (KP998809)  ...............................A..-----...........
A. pierrei (KP998810)     ..................................-----...........
A. pothieri (KP998811)    ..................................-----...........
A. ringens (KP998812)     ..................................-----...........
A. tagala (KP998814)      ..................................-----...........
A. tentaculata (KP998815) ..................................ATTAG...........
A. sp (KP998813)          ..................................-----...........

                          1        1         1         1         1         1
                          0        1         2         3         4         5
                          1        0         0         0         0         0
A. anguicida (KP998805)   CGAGTCGTTG-----AAGGAGCAATACCCAATCCTTTTGGTCTTAT----
A. gigantea (KP998806)    ..........-----...............................----
A. grandiflora (KP998807) ..........AAGGA................AT......T......----
A. kerrii (KP998808)      ..........-----........................TC.....----
A. littoralis (KP998809)  ..........-----...............................----
A. pierrei (KP998810)     ..........-----........................T...-..CAAG
A. pothieri (KP998811)    ..GT......-----........................T...-..----
A. ringens (KP998812)     T.........-----...............................----
A. tagala (KP998814)      ..........-----...............................----
A. tentaculata (KP998815) ..........-----...............................----
A. sp (KP998813)          ..........-----...............................----

                          1        1         1         1         1         2
                          5        6         7         8         9         0
                          1        0         0         0         0         0
A. anguicida (KP998805)   -CAAGAGGTGGGTATTGTTCCGCTCAATTCACTATTAATTCAATAGAGTT
A. gigantea (KP998806)    -.........................................C.......
A. grandiflora (KP998807) -T..A................A.............C......C.GT....
A. kerrii (KP998808)      -.....................T.T..........C......C..T....
A. littoralis (KP998809)  -.........................................C.......
A. pierrei (KP998810)     A..................................C......C.......
A. pothieri (KP998811)    -...A..............................C......C....T..
A. ringens (KP998812)     -..................................C......C...G...
A. tagala (KP998814)      -.................................................
A. tentaculata (KP998815) -..................................C......C.......
A. sp (KP998813)          -.................................................

                          2        2         2         2         2         2
                          0        1         2         3         4         5
                          1        0         0         0         0         0
A. anguicida (KP998805)   TTACTTCTATTGCATA------------------------------AATA
A. gigantea (KP998806)    .....CA.........------------------------------....
A. grandiflora (KP998807) .....CA...CA...G----------------------------------
A. kerrii (KP998808)      .....CA...C...A.A---------------------------AA....
A. littoralis (KP998809)  .....CA.........------------------------------....
A. pierrei (KP998810)     .....CA...C.....------------------------------....
A. pothieri (KP998811)    .....CA...C.T...ATCCACAATACAATACAATATAATATGCAT....
A. ringens (KP998812)     .....CA.........------------------------------....
A. tagala (KP998814)      ................------------------------------....
A. tentaculata (KP998815) .....C..........------------------------------....
A. sp (KP998813)          ................------------------------------....
```

	251 260 270 280 290 300
A. anguicida (KP998805)	GGCATTTT-------------------------------------TTCTT
A. gigantea (KP998806)	........ATCTA--------------------------------.AT..
A. grandiflora (KP998807)	--......AT-----------------------------------..T.A
A. kerrii (KP998808)	.......ACTCC---------------------------------..T..
A. littoralis (KP998809)	........ATCTA--------------------------------.AT..
A. pierrei (KP998810)	T.......ATCTATTT-----------------------------..T..
A. pothieri (KP998811)	T.......ATATATTTATATATATATATATATATATATATTTGTA..TG.
A. ringens (KP998812)	........ATCTA--------------------------------.AT..
A. tagala (KP998814)	........-------------------------------------.....
A. tentaculata (KP998815)	........ATCTA--------------------------------.AT..
A. sp (KP998813)	........-------------------------------------.....

	301 310 320 330 340 350
A. anguicida (KP998805)	TTTAGTTT--AAAGTTAAAAA------------GAAGT-CCA-GGGCGGA
A. gigantea (KP998806)	........--...........------------.....-...-.......
A. grandiflora (KP998807)	........--...T..T....------AAGAGGA....-A..G.......
A. kerrii (KP998808)	........--...........GAAATCAAAAAGA...G-...G.......
A. littoralis (KP998809)	........--...........------------.....-...-.......
A. pierrei (KP998810)	........AA...........------------....GG...G.......
A. pothieri (KP998811)	.......A-A......T....------------.....G...G.......
A. ringens (KP998812)	........--...........------------T....-...-.......
A. tagala (KP998814)	........--...........------------.....-...-.......
A. tentaculata (KP998815)	........--...........------------.....-...-.......
A. sp (KP998813)	........--...........------------.....-...-.......

	351 360 370 380 390 398
A. anguicida (KP998805)	TGTAGCCAAGTGGATCAAGGCAGT------------------------
A. gigantea (KP998806)	........................GGATTTGTGAATCCACCATGCGCG
A. grandiflora (KP998807)	........................GGATTTGTGAATCCACCATGCGCG
A. kerrii (KP998808)	........................GGA-TTGTGAATCCACCATGCGCG
A. littoralis (KP998809)	........................GGATTTGTGAATCCACCATGCGCG
A. pierrei (KP998810)	........................GGA-TTGTGAATCC----------
A. pothieri (KP998811)	........................GGATTTGTGAATCCACCATGCGCG
A. ringens (KP998812)	........................GGATTTGTGAATCCACCATGCGCG
A. tagala (KP998814)	........................GGATTTGTGAATCCACCATGCGCG
A. tentaculata (KP998815)	........................GGATTTGTGAATCCACCATGCGCG
A. sp (KP998813)	........................GGATTTGTGAATCCACCATGCGCG
