# Supplementary material for: DNA barcoding of Aristolochia plants and development of species-specific multiplex PCR to aid HPTLC in ascertainment of Aristolochia herbal materials
Source: PLoS One. 2018 Aug 20;13(8):e0202625. doi: 10.1371/journal.pone.0202625 (PMC6101415; doi:10.1371/journal.pone.0202625)
Supplement: S4 Fig — The numbers on the top line represent the base numbers in sequence alignment. The altered bases indicate the sequence differences. ‘.’ represents the base being identical to the first sequence. ‘–’ represents gap. (PDF) [file pone.0202625.s004.pdf]

	1 … 10 … 20 … 30 … 40 … 50
A. anguicida (KP998791)	--
A. gigantea (KP998792)	--
A. grandiflora (KP998793)	--------GGAAGGATCATTGCCGATTCCACGAAACGAGCACGACCCATG
A. kerrii (KP998794)	--
A. littoralis (KP998795)	--
A. pierrei (KP998796)	--
A. pothieri (KP998797)	GAACCTGCGGAAGGATCATTGTCGGTTCCGCGAGAGGCGCACGACCCACG
A. ringens (KP998798)	--
A. tagala (KP998800)	--
A. tentaculata (KP998801)	--
A. sp (KP998799)	--

	51 … 60 … 70 … 80 … 90 … 100
A. anguicida (KP998791)	--
A. gigantea (KP998792)	--
A. grandiflora (KP998793)	CGAACTGTATTTGCACCCCCATGTCGCCCGCTCGTGCCCCACGCCGAGTG
A. kerrii (KP998794)	--
A. littoralis (KP998795)	--
A. pierrei (KP998796)	--
A. pothieri (KP998797)	CGAACGAGTTGCGGCCGCACGGGACGCCGCGCGGCGTC------------
A. ringens (KP998798)	--
A. tagala (KP998800)	--
A. tentaculata (KP998801)	--
A. sp (KP998799)	--

	101 … 110 … 120 … 130 … 140 … 150
A. anguicida (KP998791)	--
A. gigantea (KP998792)	--
A. grandiflora (KP998793)	ACAGGCGCTGCGTTTGGGGCGGGGACTTTGCGCCGCGAGGCGCCGGGCCT
A. kerrii (KP998794)	--
A. littoralis (KP998795)	--
A. pierrei (KP998796)	--
A. pothieri (KP998797)	--
A. ringens (KP998798)	--
A. tagala (KP998800)	--
A. tentaculata (KP998801)	--
A. sp (KP998799)	--

	151 … 160 … 170 … 180 … 190 … 200
A. anguicida (KP998791)	--
A. gigantea (KP998792)	--
A. grandiflora (KP998793)	GCACTCCTTGCGCGCGTCTCGCGTCCGGACCGTCTGGACGAGCGGACGCA
A. kerrii (KP998794)	--
A. littoralis (KP998795)	--
A. pierrei (KP998796)	--
A. pothieri (KP998797)	---------GGGGGGCTCGGACGAGGGCGCCCCGCCCGCGCGGCGGCAAA
A. ringens (KP998798)	--
A. tagala (KP998800)	--
A. tentaculata (KP998801)	--
A. sp (KP998799)	--

	201 … 210 … 220 … 230 … 240 … 250
A. anguicida (KP998791)	--
A. gigantea (KP998792)	--
A. grandiflora (KP998793)	CAACACACGGCGCGATGTGCGCCAAGCGAAATGTAGAGGAAGGAGGA---
A. kerrii (KP998794)	--
A. littoralis (KP998795)	--
A. pierrei (KP998796)	--
A. pothieri (KP998797)	AAACACTCGGCGCGATCCGCGCCAAGCGAAACGCAACGGCGGGGAGGGCC
A. ringens (KP998798)	--
A. tagala (KP998800)	--
A. tentaculata (KP998801)	--
A. sp (KP998799)	--

	251 · 260 · 270 · 280 · 290 · 300
A. anguicida (KP998791)	--
A. gigantea (KP998792)	--
A. grandiflora (KP998793)	--------CTGCCGCGGCCCGGCCGCGGCGTTCGCCCCGAATTGTTAACC
A. kerrii (KP998794)	--
A. littoralis (KP998795)	--
A. pierrei (KP998796)	--
A. pothieri (KP998797)	GCCGCCGCCGCCGGTGCGGCGGCGTCCCGGATGCCCCGACGGACGGATAA
A. ringens (KP998798)	--
A. tagala (KP998800)	--
A. tentaculata (KP998801)	--
A. sp (KP998799)	--

	301 · 310 · 320 · 330 · 340 · 350
A. anguicida (KP998791)	--
A. gigantea (KP998792)	------------------------------------TGCATCGATGAAGA
A. grandiflora (KP998793)	AACACAACGACTCTCGGCAACGGATATCTTGGCTCTCGCATCGATGAAGA
A. kerrii (KP998794)	--
A. littoralis (KP998795)	--GA
A. pierrei (KP998796)	--
A. pothieri (KP998797)	ACCACCACGACTCTCGGCAACGGATATCTTGGCTCTCGCATCGATGAAGA
A. ringens (KP998798)	--GA
A. tagala (KP998800)	--
A. tentaculata (KP998801)	--GA
A. sp (KP998799)	--GA

	351 · 360 · 370 · 380 · 390 · 400
A. anguicida (KP998791)	--------------------------TAATTGCAGAA-ACCGCGAACCAT
A. gigantea (KP998792)	ACGCAGCGAAATGCGATACTTGGTGTG..........TC...........
A. grandiflora (KP998793)	ACGTAGCGAAATGCGATACTTGGTGTG..........TC...T.......
A. kerrii (KP998794)	--
A. littoralis (KP998795)	ACGCAGCGAAATGCGATACTTGGTGTG..........TC...........
A. pierrei (KP998796)	---------------------------------------...........
A. pothieri (KP998797)	ACGTAGCGAAATGCGATACTTGGTGTG..........TC...........
A. ringens (KP998798)	ACGCAGCGAAATGCGATACTTGGTGTG..........TC...........
A. tagala (KP998800)	--------------------------------------C...........
A. tentaculata (KP998801)	ACGCAGCGAAATGCGATACTTGGTGTG..........TC...........
A. sp (KP998799)	ACGCAGCGAAATGCGATACTTGGTGTG..........TC...........

	401 · 410 · 420 · 430 · 440 · 450
A. anguicida (KP998791)	CGAGTCTTTGAACGCAAGTTGCACCCGAGGCCACTAGGTCGAGGGTACAC
A. gigantea (KP998792)	....
A. grandiflora (KP998793)	.................................T................
A. kerrii (KP998794)	-.C.G..---G....-.................T....C......C....
A. littoralis (KP998795)	....
A. pierrei (KP998796)	.................................T....C.A.........
A. pothieri (KP998797)	......................G.........TG....C......C....
A. ringens (KP998798)	....
A. tagala (KP998800)	.................................T....C.A.........
A. tentaculata (KP998801)	......................................C...........
A. sp (KP998799)	....

	451 · 460 · 470 · 480 · 490 · 500
A. anguicida (KP998791)	CTGCCTGGGCGTCACGCCCGAC--GTGTTCT-CCCCCCACCCCGCGCGCC
A. gigantea (KP998792)	......................--...C...-......G..........T
A. grandiflora (KP998793)	......................--.......C....---..T.....A--
A. kerrii (KP998794)	......................--.CC....-......G........T.G
A. littoralis (KP998795)	......................--...C...C......G..........T
A. pierrei (KP998796)	......................--.C.....-.T....G........A--
A. pothieri (KP998797)	......................--.C.....C....A.G..T.-------
A. ringens (KP998798)	......................--.C.C...-......G........C..
A. tagala (KP998800)	......................--.C.....-......G........T--
A. tentaculata (KP998801)	......................GT.......C.....AC...T....C.T
A. sp (KP998799)	......................--.......-..................

	501 510 520 530 540 550
A. anguicida (KP998791)	CCCGCCCCGCCGCGGGCGGCGCCGCGCGTCG-ACGGGGGCGAGCAGATTG
A. gigantea (KP998792)	........AT............G........-..................
A. grandiflora (KP998793)	-----------......AC.C.......C---G.TC..............
A. kerrii (KP998794)	.----------------------........CC.............-...
A. littoralis (KP998795)	........A............GG........-..................
A. pierrei (KP998796)	--------------------..G........-T.............GA..
A. pothieri (KP998797)	-----...TT......G.AT..G.--------G...-----.....T...
A. ringens (KP998798)	.......AA........C....G........-..................
A. tagala (KP998800)	--------------------........G..-G.............GC..
A. tentaculata (KP998801)	......G.AG......TC.T..G........-T..............G..
A. sp (KP998799)	...............................-..................

	551 560 570 580 590 600
A. anguicida (KP998791)	GCCGTCCCTG--CCCCCC--GGGGCGAGGTTGGCTG-AAAAACGC-TGTG
A. gigantea (KP998792)	.T.A......--......--................A.....T..-.T..
A. grandiflora (KP998793)	..........--......CG..........C....T-...T.----.CA.
A. kerrii (KP998794)	..........CC.....TCC..........C...CTA...GGAATCG.A.
A. littoralis (KP998795)	.T........--......--................A.....T..-.T..
A. pierrei (KP998796)	........C.CC......GG..T.......C...CTA...GG.AA----.
A. pothieri (KP998797)	........C.-C....G.GA..........C...CCA...GC..G----.
A. ringens (KP998798)	.T..C.....--......--................-........-.T..
A. tagala (KP998800)	..........-G......GG..T.......C....TA...GG.AA----.
A. tentaculata (KP998801)	.T........--......--................-...TG...T.T..
A. sp (KP998799)	..........--......--................-........-....

	601 610 620 630 640 650
A. anguicida (KP998791)	C-CCCGC--GGGCACTTCGGCACGACGAGTGGTGGCTCCGCCCT-CCCCG
A. gigantea (KP998792)	.-...A.--...................................-...T.
A. grandiflora (KP998793)	.-.....--....T.-A....G....T...........G.....-.....
A. kerrii (KP998794)	AG.....GC....T.-G....G....CGT.........GAAG..C.....
A. littoralis (KP998795)	.-...A.--...................................-.....
A. pierrei (KP998796)	.-...A.--....T.-G....G....T.C..........A.G.AC.....
A. pothieri (KP998797)	.-...C.GG....T.-G....G....TGC.........GATG..------
A. ringens (KP998798)	.-...A.--...................................-.....
A. tagala (KP998800)	.-...A.--....T.-G....G....T.C..........A.G..C.....
A. tentaculata (KP998801)	.-...A.--....G..G...........................-.....
A. sp (KP998799)	.-.....--...................................-.....

	651 660 670 680 690 700
A. anguicida (KP998791)	GCC-CT-GCGTGGCTCGAAGTCGTGTCCGAGGCC-------------GGG
A. gigantea (KP998792)	...-..-...........................-------------...
A. grandiflora (KP998793)	...TT.-.TTA..................T.....CCCG----TTGA...
A. kerrii (KP998794)	...C.CA..CG...C........C.C...C.T...CCCCGCTCGGGAA..
A. littoralis (KP998795)	...-..-...........................-------------...
A. pierrei (KP998796)	...TTG-..CG...C........C.CA..C.AA..-CCCCGCTGGGAA.T
A. pothieri (KP998797)	-------..CG..GG........C.AT..C.AA..--CCGGCCGGGAA..
A. ringens (KP998798)	...-..-...........................-------------A..
A. tagala (KP998800)	...TTG-..CG...C........C.C...C.AA..-CCCCGCTGGAAA..
A. tentaculata (KP998801)	...-..-...........................-------------...
A. sp (KP998799)	...-..-...........................-------------...

	701 710 720 730 740 750
A. anguicida (KP998791)	C-TGTGAAGAAGGACCCCTGCCGGCAGTCGCCTC---CGCGAGC--GGA-
A. gigantea (KP998792)	.-.CC.......C..............C......---.......GC...-
A. grandiflora (KP998793)	.-.AC.----..C.....C......T.CT.....CGGT...CA.T-...-
A. kerrii (KP998794)	.-CCC.-..G........C......C.CTT.GC.CTC...CGT.TC...-
A. littoralis (KP998795)	.-.CC......................C......CGC.......--...-
A. pierrei (KP998796)	.-..C.----...............T.C...GC.TCCT.T.TC---...-
A. pothieri (KP998797)	.-C.C.----.......AATG....GCC.CG.C.---..G.GA.CC..GG
A. ringens (KP998798)	.-.CC.C...........................---.......--...-
A. tagala (KP998800)	.-..C.----...............T.C...GC.---TC..GTATC...-
A. tentaculata (KP998801)	.A.T...GAG........................---..T.GT.--...-
A. sp (KP998799)	.-................................---.......--...-

Species	751–800 (markers at 751, 760, 770, 780, 790, 800)
A. anguicida (KP998791)	-----GGCCT--TGCTCGGATTGCGACCCCAGGTCAGGTGGGAACACCCG
A. gigantea (KP998792)	-----.....-T......................................
A. grandiflora (KP998793)	-----..AGC-C.............................T......
A. kerrii (KP998794)	-----...GCGC.......A................-------------
A. littoralis (KP998795)	-----....C-T......................................
A. pierrei (KP998796)	-----T..GCGC.......A......................T......
A. pothieri (KP998797)	AGGGT...GCGCC......A......................T......
A. ringens (KP998798)	-----.....-C......................................
A. tagala (KP998800)	-----T..GCGC.......A......................T......
A. tentaculata (KP998801)	-----.....--C......A..............................
A. sp (KP998799)	-----.....--......................................

Species	801–835 (markers at 801, 810, 820, 830, 835)
A. anguicida (KP998791)	CTGAGTTTAAGCATATCA-----------------
A. gigantea (KP998792)	..................ATAAGCGGAGGAAATCG
A. grandiflora (KP998793)	..................ATA--------------
A. kerrii (KP998794)	--TG..AC......G--------------------
A. littoralis (KP998795)	..................ATA--------------
A. pierrei (KP998796)	..................-----------------
A. pothieri (KP998797)	..................ATA--------------
A. ringens (KP998798)	..................ATA--------------
A. tagala (KP998800)	.................------------------
A. tentaculata (KP998801)	..................ATA--------------
A. sp (KP998799)	..................ATA--------------
