# Supplementary material for: DNA barcoding of Aristolochia plants and development of species-specific multiplex PCR to aid HPTLC in ascertainment of Aristolochia herbal materials
Source: PLoS One. 2018 Aug 20;13(8):e0202625. doi: 10.1371/journal.pone.0202625 (PMC6101415; doi:10.1371/journal.pone.0202625)
Supplement: S5 Fig — Arrows indicate orientation and position of the primer set of multiplex PCR. (PDF) [file pone.0202625.s005.pdf]

Taxon	351 · 360 · 370 · 380 · 390 · 400 · 410 (ITS-Aris-390F →)
A. anguicida (KP998791)	---------------TAATTGCAGAA-ACCGCGAACCATCGAGTCTTTGAACGCAAGTTG
A. gigantea (KP998792)	TGCGATACTTGGTGTG..........TC................................
A. grandiflora (KP998793)	TGCGATACTTGGTGTG..........TC...T............................
A. kerrii (KP998794)	--.C.G..---G..........
A. littoralis (KP998795)	TGCGATACTTGGTGTG..........TC................................
A. pierrei (KP998796)	---------------------------.................................
A. pothieri (KP998797)	TGCGATACTTGGTGTG..........TC................................
A. ringens (KP998798)	TGCGATACTTGGTGTG..........TC................................
A. tagala (KP998800)	---------------------------C................................
A. tentaculata (KP998801)	TGCGATACTTGGTGTG..........TC................................
A. sp (KP998799)	TGCGATACTTGGTGTG..........TC................................
R. pulchellum (MG870094)	TGCGATAGTTGGTGTG..........TC...T.......G....................
G. integrifolium (MG870090)	TGCGATACTTGGTGTG........G.TC..........C.....................
J. sambac (MG870093)	TGCGATACTTGGTGTG..........TC...T............T...............
J. adenophyllum (MG870091)	TGCGATACTTGGTGTG..........TC...T............T...............
J. sp (MG870092)	TGCGATACTTGGTGTG..........TC...T............T...............

Taxon	411 · 420 · 430 · 440 · 450 · 460 · 470
A. anguicida (KP998791)	CACCCGAGGCCACTAGGTCGAGGGTACACCTGCCTGGGCGTCACGCCCGACGTGTTCTCC
A. gigantea (KP998792)	....C.....
A. grandiflora (KP998793)	............T...
A. kerrii (KP998794)	............T....C......C...........................CC......
A. littoralis (KP998795)	....C.....
A. pierrei (KP998796)	............T....C.A................................C......T
A. pothieri (KP998797)	.G.........TG....C......C...........................C.......
A. ringens (KP998798)	....C.C.....
A. tagala (KP998800)	............T....C.A................................C.......
A. tentaculata (KP998801)	.................C.....................................GT..T
A. sp (KP998799)	....
R. pulchellum (MG870094)	.G.....A....T....C......C..GT.................ATTG...CG..C..
G. integrifolium (MG870090)	.G....GA...TTCT..C......C..GT.................ATCG.---.G.C..
J. sambac (MG870093)	.G.....A...T..GT.CT.....C..G..................ATC....CGC.CTT
J. adenophyllum (MG870091)	.G.....A...T..GT.CT.....C..GT.................ATC....CGC.CT.
J. sp (MG870092)	.G.....A...T..GT.CT.....C..G..................ATC....CGC.CTT

Taxon	471 · 480 · 490 · 500 · 510 · 520 · 530
A. anguicida (KP998791)	CCCC-----ACCCCGCGCGCCCCCGCCCCGCCGCGGGCGGCGCCG--CGCGTCG-ACGGG
A. gigantea (KP998792)	....-----G..........T........AT............G.----------.....
A. grandiflora (KP998793)	....-------.T.....A--------------.....AC.C.........C---G.TC.
A. kerrii (KP998794)	....-----G........T.G.G------------------------------.CC....
A. littoralis (KP998795)	....C----G..........T........A............GG----------......
A. pierrei (KP998796)	....-----G........A-----------------------.G.----------T....
A. pothieri (KP998797)	...AC----G..T--------------..TT......G.AT..G.----------G....
A. ringens (KP998798)	....-----G........C.........AA........C....G.----------.....
A. tagala (KP998800)	....-----G........T------------------------------..G.--G....
A. tentaculata (KP998801)	....CCA--C...T....C.T......G.AG......TC.T..G.----------T....
A. sp (KP998799)	....-----....................................--.......-.....
R. pulchellum (MG870094)	....GCCT-GA....T.TC.T------------AA..G.....G.TC.T.AG..TG....
G. integrifolium (MG870090)	...ACGCAAC....A.T...-----------G.TTT..T....G-----------G....
J. sambac (MG870093)	T.G.CACTGG....A.--------------------------GGA--TC.A...-G..TY
J. adenophyllum (MG870091)	.G..ACCAGG...TTT.-------------------------GGA--TCG....-G..TT
J. sp (MG870092)	T.G.CACTGG....A.--------------------------GGA--TC.A...-G..TY

Taxon	531 · 540 · 550 · 560 · 570 · 580 · 590 (ITS2-taF →)
A. anguicida (KP998791)	GGCGAGCAGATTGGCCGTCCCTG----CCCCCC---GGGGCGAGGTTGGCTGAAAAACG-
A. gigantea (KP998792)	..............T.A......----......---.....................AT-
A. grandiflora (KP998793)	.......................----......CG-..........C....T...T.TC-
A. kerrii (KP998794)	.......................CCCC...T..---..........C...CT....GGAA
A. littoralis (KP998795)	..............T........----......---.....................AT-
A. pierrei (KP998796)	.........GA..........C.---C......CGG..T.......C...CT....GGC-
A. pothieri (KP998797)	.-----...T...........C.--------.GCGA..........C...CC....G.C-
A. ringens (KP998798)	..............T..C.....----......---.......................-
A. tagala (KP998800)	.........GC............----G.....CGG..T.......C....T....GGC-
A. tentaculata (KP998801)	..........G...T........----......---...................TG..-
A. sp (KP998799)	.......................----......---.......................-
R. pulchellum (MG870094)	..G.C.G.AG.....TTC..G..--------AG---T.TTT.C..C.A..CTG...CACG
G. integrifolium (MG870090)	...--A..CGC.....TC..G..----.G.A..---.TC...C..A.....T...TT..-
J. sambac (MG870093)	..GTT.G.AG......TC..G..----.G...G---.CC...C..C....CC..------
J. adenophyllum (MG870091)	..GCT.G.AG......TC..G..----.G..TG---.CT.T.C.......CC...TG..-
J. sp (MG870092)	..GTT.G.AG......TC..G..----.G...G---.CC...C..C....CC..------

Taxon	591 · 600 · 610 · 620 · 630 · 640 · 650 (ITS2-piF →)
A. anguicida (KP998791)	--CTGTGCCCCGC--GGGCACTTCGGCACGACG----AGTGGTGGCTCCGCCCTCCCCGG
A. gigantea (KP998792)	-G..T......A.--..................----....................T..
A. grandiflora (KP998793)	-----A.......--....T.-A....G....T----...........G...........
A. kerrii (KP998794)	TCGGAGAG.....GC....T.-G....G....C----GT.........GAAG......C.
A. littoralis (KP998795)	-G..T......A.--..................----.......................
A. pierrei (KP998796)	----AA.....A.--....T.-G....G....T----.C..........A.G.A....C.
A. pothieri (KP998797)	----.G.....C.GG....T.-G....G....T----GC.........GATG.-------
A. ringens (KP998798)	----T......A.--..................----.......................
A. tagala (KP998800)	----AA.....A.--....T.-G....G....T----.C..........A.G......C.
A. tentaculata (KP998801)	-CT.T......A.--....G.-G..........----.......................
A. sp (KP998799)	--...........--..................----.......................
R. pulchellum (MG870094)	GTTCCCT.G....---------A..T.G....A----........TC-------------
G. integrifolium (MG870090)	-----A.T..TCG--.C..CTG...T.G....ACTACG.....T.....AA..-------
J. sambac (MG870093)	--TC.GAT..G..ACT...G.-GT.CTT....A----.T......T---.AA..A.T.AA
J. adenophyllum (MG870091)	------AT..G..ACT.A.G.-GT.C.T....A----.T......T---.AA..A.T.AA
J. sp (MG870092)	--TC.GAT..G..ACT...G.-GT.CTT....A----.T......T---.AA..A.T.AA

Taxon	651 … 660 … 670 … 680 … 690 … 700 … 710
A. anguicida (KP998791)	CCCT---GCGTGGCTCGAAGTCGTGTCCGAGGCC------------------CGGGC-TG
A. gigantea (KP998792)	....---...........................------------------.....-.C
A. grandiflora (KP998793)	..T.T--.TTA..................T...-----------CCCCGTTGA....-.A
A. kerrii (KP998794)	G..CCCA..CG...C........C.C...C.T..------CCCCCGCTCGGGAA...-CC
A. littoralis (KP998795)	....---...........................------------------.....-.C
A. pierrei (KP998796)	G...TG-..CG...C........C.CA..C.AA.-------CCCCCGCTGGGAA.T....
A. pothieri (KP998797)	-------..CG..GG........C.AT..C.AA.--------CCCGGCCGGGAA...-C.
A. ringens (KP998798)	....---...........................-------------------A...-.C
A. tagala (KP998800)	G...TG-..CG...C........C.C...C.AA.-------CCCCCGCTGGAAA......
A. tentaculata (KP998801)	....---...........................------------------.....A.T
A. sp (KP998799)	....---...........................------------------.....-..
R. pulchellum (MG870094)	------------.TCTT.GA.T..CG...TTG--------C--------CGG.A......
G. integrifolium (MG870090)	------------.TA.CGC....C.A..TCA...CGCACACCTCCTCCCCTG..A...--
J. sambac (MG870093)	.TT---------..GT.TT...--------------------------------------
J. adenophyllum (MG870091)	.TT---------..GT.TT....A..T.T..CG.--------------------------
J. sp (MG870092)	.TT---------..GT.TT...--------------------------------------

ITS2-poF (711 → 740)

Taxon	711 … 720 … 730 … 740 … 750 … 760 … 770
A. anguicida (KP998791)	TGAAGAAGGA-----------CCCCTGCCGGCA-GTCGCCTC-------CGCGAGC---G
A. gigantea (KP998792)	C.......C.-----------............-.C......-------.......GC-.
A. grandiflora (KP998793)	C.......C.-----------....C......T-.CT.....CG------.T.C..ACT.
A. kerrii (KP998794)	C..G......-----------....C......C-.CTT.GC.CT----C...CGT.TC-.
A. littoralis (KP998795)	C.........-----------............-.C......CG----C.......---.
A. pierrei (KP998796)	C.........-----------...........T-.C...GC.TC-----.TGTGT.....
A. pothieri (KP998797)	C.........-----------...AATG....GCCC....C.GGGGACC..G.GAG---.
A. ringens (KP998798)	C.C.......-----------............-........-------.......---.
A. tagala (KP998800)	C.........-----------...........T-.C...GC.TC-----..GT.T.....
A. tentaculata (KP998801)	...GAG....-----------............-........-------..T.GT.....
A. sp (KP998799)	..........-----------............-........-------.......---.
R. pulchellum (MG870094)	C.TC..G..GAGCATTTGGAC..TG...GT..GA.CT.AGC.CTTCGCCTT..GCG---A
G. integrifolium (MG870090)	-..GCG....------------T...T-------.C..A.C.TCTGAA-..TCGT.CCCA
J. sambac (MG870093)	------..AG------------T..A..GC..CAT.G..T.TCCGGATG..TTG..CCTC
J. adenophyllum (MG870091)	----------------------.......A.------..T.TCCGGATG..TTG..CCTC
J. sp (MG870092)	------..AG------------T..A..GC..CAT.G..T.TCCGGATG..TTG..CCTC

Taxon	771 … 780 … 790 … 800 … 810 … 820 … 830
A. anguicida (KP998791)	GAGGCCT--TGCTC---GGATTGCGACCCCAGGTCAGGTGGGAACACCCGCTGAGTTTAA
A. gigantea (KP998792)	.......-T.....---...
A. grandiflora (KP998793)	....AGC-C.....---..........................T................
A. kerrii (KP998794)	.....GCGC.....---...A................---------------TG..AC..
A. littoralis (KP998795)	......C-T.....---...
A. pierrei (KP998796)	..T..GCGC.....---...A......................T................
A. pothieri (KP998797)	.T...GCGCC....---...A......................T................
A. ringens (KP998798)	.......-C.....---...
A. tagala (KP998800)	..T..GCGC.....---...A......................T................
A. tentaculata (KP998801)	.......--C....---...A.......................................
A. sp (KP998799)	.......--.....---...
R. pulchellum (MG870094)	.G...GAAC.C.AA---C.G..................C...GCT...............
G. integrifolium (MG870090)	A..A.GA--.....---TCGAC................C....CT...............
J. sambac (MG870093)	AC..TGCACC..A.CCTC....................C....CT...............
J. adenophyllum (MG870091)	AC..TGCGCC..A.CTTC..C.................C....CT...............
J. sp (MG870092)	AC..TGCACC..A.CCTC....................C....CT...............

ITS4 (← 831–850)

Taxon	831 … 840 … 850 … 855
A. anguicida (KP998791)	GCATATCA-----------------
A. gigantea (KP998792)	........ATAAGCGGAGGAAATC G
A. grandiflora (KP998793)	........ATA--------------
A. kerrii (KP998794)	....G--------------------
A. littoralis (KP998795)	........ATA--------------
A. pierrei (KP998796)	........-----------------
A. pothieri (KP998797)	........ATA--------------
A. ringens (KP998798)	........ATA--------------
A. tagala (KP998800)	.......------------------
A. tentaculata (KP998801)	........ATA--------------
A. sp (KP998799)	........ATA--------------
R. pulchellum (MG870094)	........A----------------
G. integrifolium (MG870090)	........A----------------
J. sambac (MG870093)	........ATAA-------------
J. adenophyllum (MG870091)	........ATAA-------------
J. sp (MG870092)	........A----------------
